# Novel Locally Active Estrogens Accelerate Cutaneous Wound Healing-Part 2

**DOI:** 10.1038/s41598-017-02820-y

**Published:** 2017-05-31

**Authors:** Mario Brufani, Nicoletta Rizzi, Clara Meda, Luigi Filocamo, Francesca Ceccacci, Virginia D’Aiuto, Gabriele Bartoli, Angela La Bella, Luisa M. Migneco, Rinaldo Marini Bettolo, Francesca Leonelli, Paolo Ciana, Adriana Maggi

**Affiliations:** 1 Dipartimento di Scienze Biochimiche “A. Rossi Fanelli”, Universita‘ degli Studi di Roma “La Sapienza”, Via degli Apuli 9, I-00185 Roma, Italy; 2 Dipartimento di Chimica “S. Cannizzaro”, Universita‘ degli Studi di Roma “La Sapienza”, P.le Aldo Moro 5, I-00185 Roma, Italy; 3 Dipartimento di Biologia Ambientale, Universita‘ degli Studi di Roma “La Sapienza”, P.le Aldo Moro 5, I-00185 Roma, Italy; 4 Istituto di Metodologie Chimiche-CNR, Unità Organizzativa di Supporto, Sede di Roma, Università degli Studi di Roma “La Sapienza”, P. le Aldo Moro, 5, I-00185 Roma, Italy; 5 Centre of Excellence on Neurodegenerative Diseases, Universita‘ degli Studi di Milano, Via Balzaretti, 9, I-20133 Milano, Italy

## Abstract

Estrogen deprivation is associated with delayed healing, while estrogen replacement therapy (ERT) accelerates acute wound healing and protects against development of chronic wounds. However, current estrogenic molecules have undesired systemic effects, thus the aim of our studies is to generate new molecules for topic administration that are devoid of systemic effects. Following a preliminary study, the new 17β-estradiol derivatives **1** were synthesized. The estrogenic activity of these novel compounds was evaluated *in vitro* using the cell line ERE-*Luc* B17 stably transfected with an ERE-*Luc* reporter. Among the 17β-estradiol derivatives synthesized, compounds **1e** and **1f** showed the highest transactivation potency and were therefore selected for the study of their systemic estrogenic activity. The study of these compounds in the ERE-*Luc* mouse model demonstrated that both compounds lack systemic effects when administered in the wound area. Furthermore, wound-healing experiments showed that **1e** displays a significant regenerative and anti-inflammatory activity. It is therefore confirmed that this class of compounds are suitable for topical administration and have a clear beneficial effect on wound healing.

## Introduction

Large experimental evidence demonstrated that the sex steroid estrogen protects against developing a chronic wound^1^, delays delayed healing, particularly in the elderly^2^, and estrogen replacement accelerates healing in aged humans and hormone-deprived animal models^3, 4^. Several studies addressed the mechanisms underlying such an effect and showed that estrogenic compounds play a prominent role in promoting the healing processes by modulating the inflammatory response, accelerating re-epithelialization, inducing granulation, and modifying proteolysis in skin cells, especially keratinocytes^5, 6^.

Recent studies provided new insights on the molecular mechanisms mediating the estrogen protective function. It is now known that macrophages and neutrophils express the intracellular receptors for estrogens (ERα and ERβ) and in these cells the 17β-estradiol-ER complex may exert anti-inflammatory activity by inhibiting NF-kB nuclear translocation^7^ and by accelerating macrophage transition from the inflammatory phase to the so called M2 polarization stage that is involved in tissue repair and reconstruction^8^.

Considering the necessity to find efficacious therapeutic means for healing of wounds, particularly in the elder population, estrogens should be taken in consideration, yet the serious, undesired side effects, particularly cancer promotion, associated with the use of these hormones prevents their therapeutic application. Therefore, to exploit the beneficial effects of estrogens on wound healing, while avoiding their undesired side effects, we attempted to obtain locally active estrogens.

Some years ago we reported the synthesis and pharmacological activity of alkyl ester **2**, derivatives of 17β-estradiol at C-17^9^.

**Figure 1:**
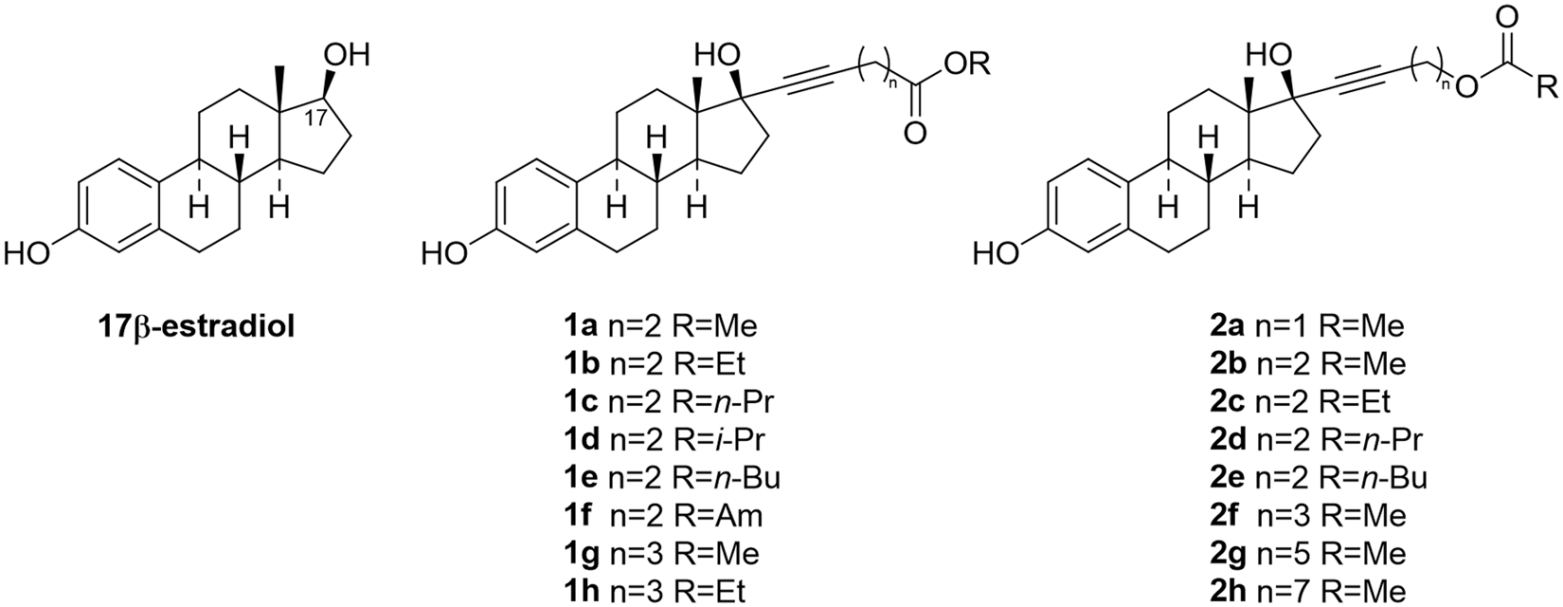
Chemical structures of 17β-estradiol and derivatives 1 and 2.

In these compounds the metabolite resulting from the hydrolysis of the ester group is an alcohol that can quickly undergo metabolic oxidation, and therefore further loses affinity for ERs^10–13^. In fact, compound **2e**, with the highest transactivation potency, showed *in vivo* substantially accelerated healing without systemic activity. Continuing these studies, we decided to generate a series of novel ester derivatives of 17β-estradiol at C-17 in which the metabolic hydrolysis would generate directly a carboxylic acid (Fig. 1).

We here show the studies carried out with topic application of these compounds that demonstrate their efficacy in wound healing and their lack of effects at systemic level.

## Results and Discussion

### Synthesis of Compounds 1

**Figure 2:**
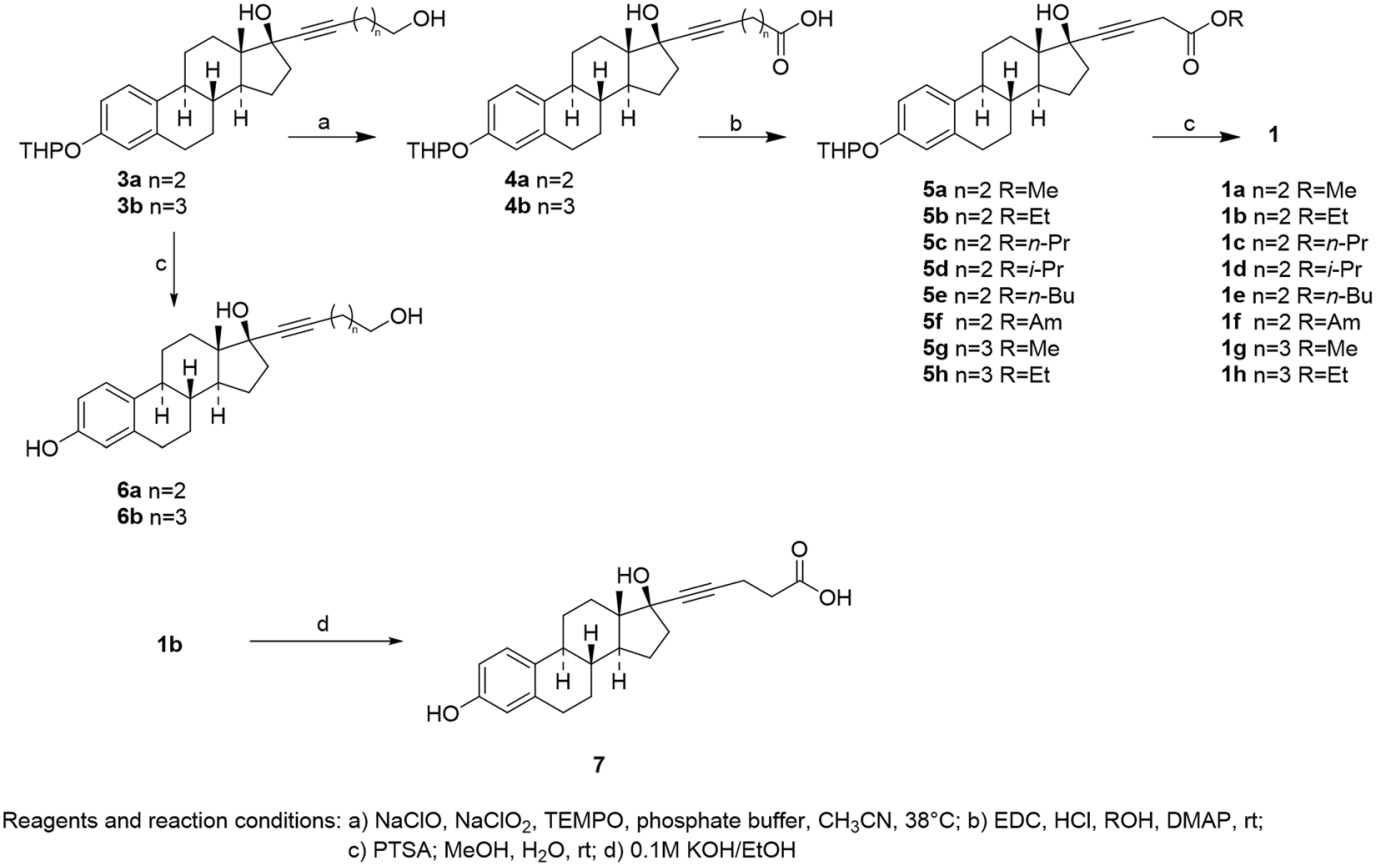
Reagents and reaction conditions. (**a**) NaClO, NaClO_2_, TEMPO, Phosphate buffer, CH_3_CN, 38 °C. (**b**) EDC, HCl, ROH, DMAP, rt. (**c**) PTSA, MeOH, H_2_O, rt. (**d**) 0.1 M KOH/EtOH.

The starting material for the synthesis of compounds **1** were the known 17β-estradiol derivatives **3**, whose preparation we have previously described^9^. The first step in the synthesis was conversion of **3** to the corresponding carboxylic acids **4** by means of a 2,2,6,6-tetramethylpiperidinyloxy (TEMPO) catalyzed oxidation, using NaClO_2_ as stoichiometric oxidant (Fig. 2)^14^.

Compounds **4** were then converted into compounds **5** by esterification with a number of alcohols in the presence of 1-ethyl-3-(3-dimethylaminopropyl)carbodiimide hydrochloride (EDC) and 4-dimethylaminopyridine (DMAP)^15^. Deprotection of the phenol function with *p*-toluensulphonic in a MeOH/H_2_O mixture gave the target compounds **1**. Compounds **3** were eventually unprotected with *p*-toluensulphonic in a MeOH/H_2_O mixture to give 17β-estradiol derivatives **6**. Finally, **1b** was submitted to basic hydrolysis to give **7(H-NMR and C-NMR spectra of new 17﻿β﻿-estradiol derivatives 1, 6 and 7 are shown in Supplementary Information)**.

### Estrogenic Activity of the New Chemical Entities (NCE)

#### *In vitro* studies - cell transfection assay

**Figure 3:**
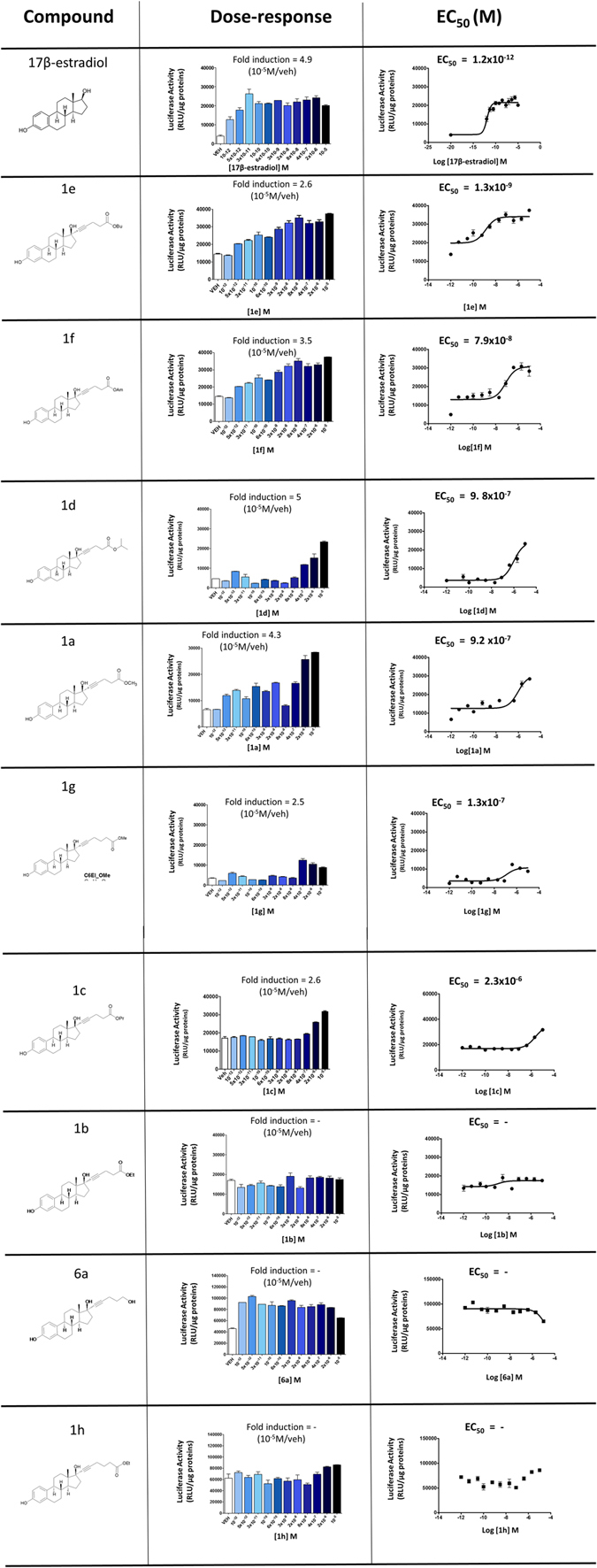
Compounds: chemical structure of tested compounds. Dose-response: ER transcriptional activity measured as luciferase activity in the presence of increasing concentrations of indicated compounds in the ERE-Luc B17 cells, fold induction of luciferase activity measured at 10-5 M of indicated compound respect to vehicle. EC50: plotting of the transactivation data for EC50 calculation by means of sigmoidal dose-response (variable slope) using PRISM5 software.

The ability of the novel compounds to transcriptionally activate ERs was tested in ERE-*Luc B*_*17*_ cells, a clone of the breast cancer cell line MCF-7 stably transfected with a reporter constituted by the luciferase gene driven by an estrogen-regulated synthetic promoter previously generated and tested in our laboratories^16^. Several compounds displayed a transactivation potency (EC_50_) of an order of magnitude compatible for therapeutic use as most synthesized compounds had an EC_50_ between 2.3 × 10^−6^ and 9.8 × 10^−7^ M. The highest transactivation activity was shown by compound **1e** and **1f** with an EC_50_ of 1.3 × 10^−9^ and 7.9 × 10^−8^ M, respectively. Compounds **1b**, **6a** and **1h** did not show any activity on ERs (Fig. 3).

The compounds **1e** and **1f** were therefore selected for a further study to evaluate their estrogenic activity *in vivo*.

#### *In vivo* imaging

To investigate the activity of **1e** and **1f** at systemic level, we utilized the ERE-*Luc* reporter mice. The ERE-*luc* is a transgenic model obtained by random integration of a luciferase reporter driven by an estrogen-responsive promoter. The ubiquitous, regulated expression of luciferase was obtained by flanking the reporter construct with insulators^15^. A large number of experimental evidence demonstrates that in this mouse luciferase activity is strictly correlated with the state of ER transcriptional activity^16–18^ and that the sensitivity of the response to estrogen is sufficient to measure by total body *in vivo* bioluminescence the subtle physiological changes in ER activity typical of the different phases of the reproductive cycle.

**Figure 4:**
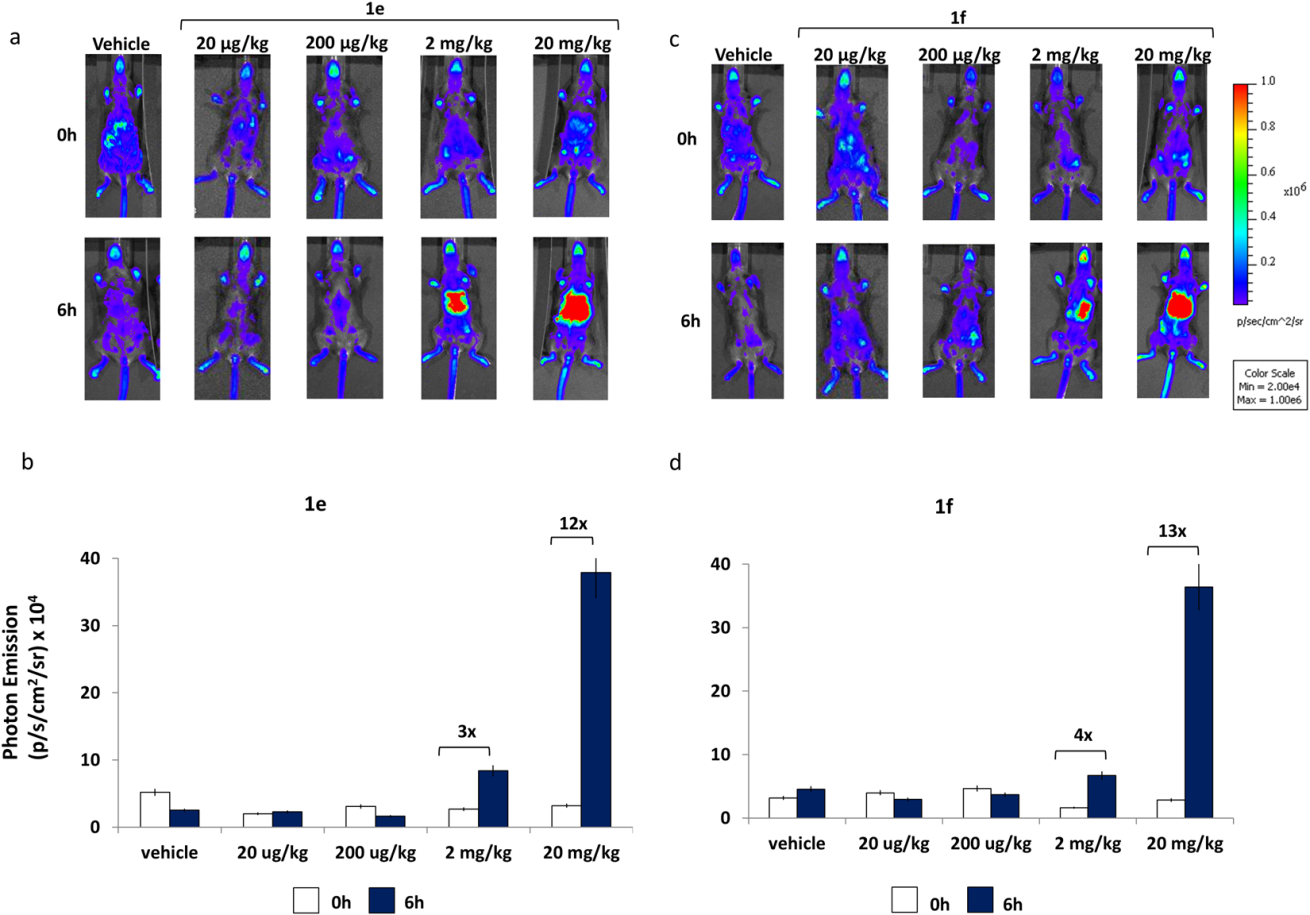
Effect of NCE on ER transcriptional activity in living ERE-Luc mice: dose- response. (**a**) Optical imaging of ERE-Luc mice at 0 and 6 hours after treatment with the indicated dosages of **1e** compound. Photos portray whole body (ventral view) of a representative individual in a pseudocolor image indicating the intensity of photon emission from the different body areas. (**b**) *In vivo* BLI: quantification of photon emission whole body at 0 and 6 hours after injection of **1e** at indicated dosages. (**c**) Optical imaging of ERE-Luc mice at 0 and 6 hours after treatment with the indicated dosages of **1f** compound. Photos portray whole body (ventral view) in a pseudocolor image processed to indicating the intensity of photon emission from the different body areas of a representative animal for each group. (**d**) Whole body photon emission quantification at 0 and 6 hours after **1f** treatment at indicated doses. Bars correspond to mean +/− SEM (n = 4).

Here, the first study we carried out was a large-range dose-response analysis to measure whole body luciferase assay after subcutaneous administration of: (1) the vehicle; (2) corn oil as control; (3) the two compounds **1e** and **1f** at the dosage of 20 μg/kg, 200 μg/kg, 2 mg/kg and 20 mg/kg. The mice (4 for each experimental group) were subjected to *in vivo* whole body imaging 6 h after the pharmacological treatment. This time was selected on the basis of previous studies showing that the peak of ER transcriptional activation generally occurs at 6–8 h after administration of the estrogenic compounds^16, 17^. The administration of 20 and 200 μg/kg of **1e** and **1f** did not induce any measurable effect at systemic level, at the higher dosages (2 and 20 mg/kg) both compound **1e** and **1f** induced the expression of the luciferase reporter around the area of subcutaneous injection and in some other body areas (Fig. 4).

This observation suggested that **1e** and **1f** up to the dose of 200 μg/kg acted locally by showing some activity at the administration site, but not a systemic level and led us to further evaluate their effects in a wound healing model.

#### Properties of **1e** and **1f** when administered in the area surrounding a wound

To study the estrogenic and healing properties of the two compounds, we generated a wound in the ERE-*Luc* mouse model and tested the activity of the two compounds administered s.c. in the area surrounding the wound as described in the methodology section.

**Figure 5:**
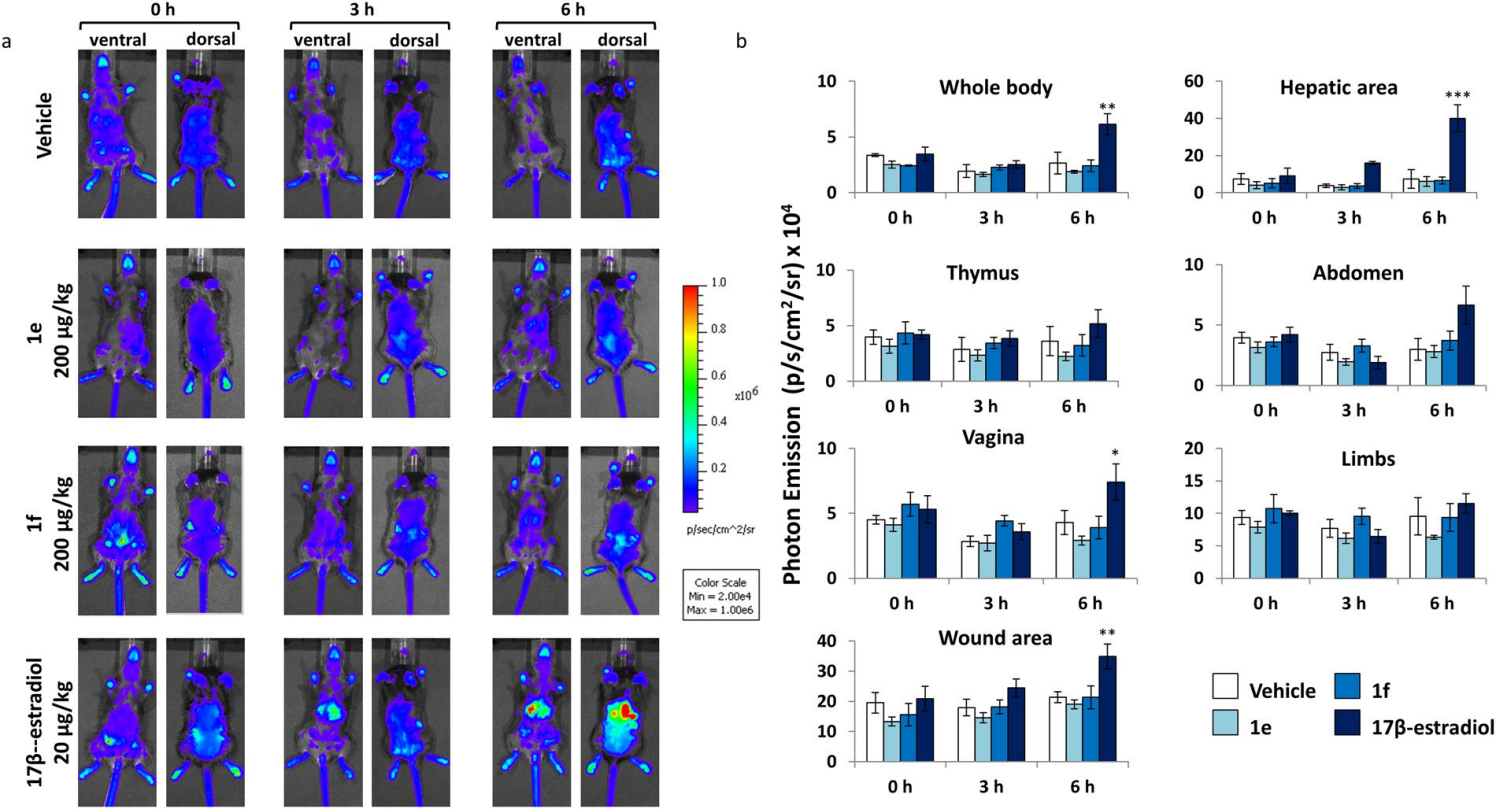
Time-course analysis of ER activity in selected body areas after administration of synthetic and natural estrogens. (**a**) Optical imaging of ERE-*Luc* mice at 0, 3 and 6 hours after the s.c. injection of the indicated compounds. Photos portray whole body (ventral and dorsal view) pseudocolor images representing the amount of photon emission. Each individual is representative of its own group. (**b**) *In vivo* BLI: photon emission from selected body areas were measured. Bars represent the mean +/− SEM of photon emission measured in 4 animals/group. *p < 0.05; **p < 0.01; ***p < 0.001 vs time 0 ANOVA followed by Bonferroni’s test for multiple comparisons.

The study was done in the following experimental groups: (1) vehicle (corn oil), (2) **1e** (200 µg/kg s.c.); (3) **1f** (200 µg/kg s.c.) and 4) 17β-estradiol (20 µg/kg s.c.) as positive control. Each group was composed of 4 ERE-*Luc* females’ mice ovariectomized 2 weeks’ prior the experiment to avoid any interference due to endogenous estrogens. Photon emission was measured in the living animals three times: at 0, 3 and 6 h after s.c. injection. Figure 5 shows the images of the mice in pseudocolors as obtained by the CCD camera in panel a and the measurement of the amount of photon emission by whole body, thymus, hepatic area, abdomen, limbs, vagina (ventral view) and in wound area (dorsal view) in panel b.

Most relevant was the fact that we did not observe increased photon emission in any of the body areas taken into consideration at 3 or 6 h after the topic administration of **1e** and **1f**; in contrast, 17β-estradiol treatment induced a significant increase of luciferase activity in the whole body and in several of the body areas analyzed. In the wound area photon emission measured at 6 hours time showed a trend to increase in animals treated with both **1e** and **1f** (30% and 20% increase with respect to time 0, respectively). In this area 17β-estradiol induced a 40% increase with respect to vehicle-treated mice; no increase was registered in the animals treated with the vehicle.

**Figure 6:**
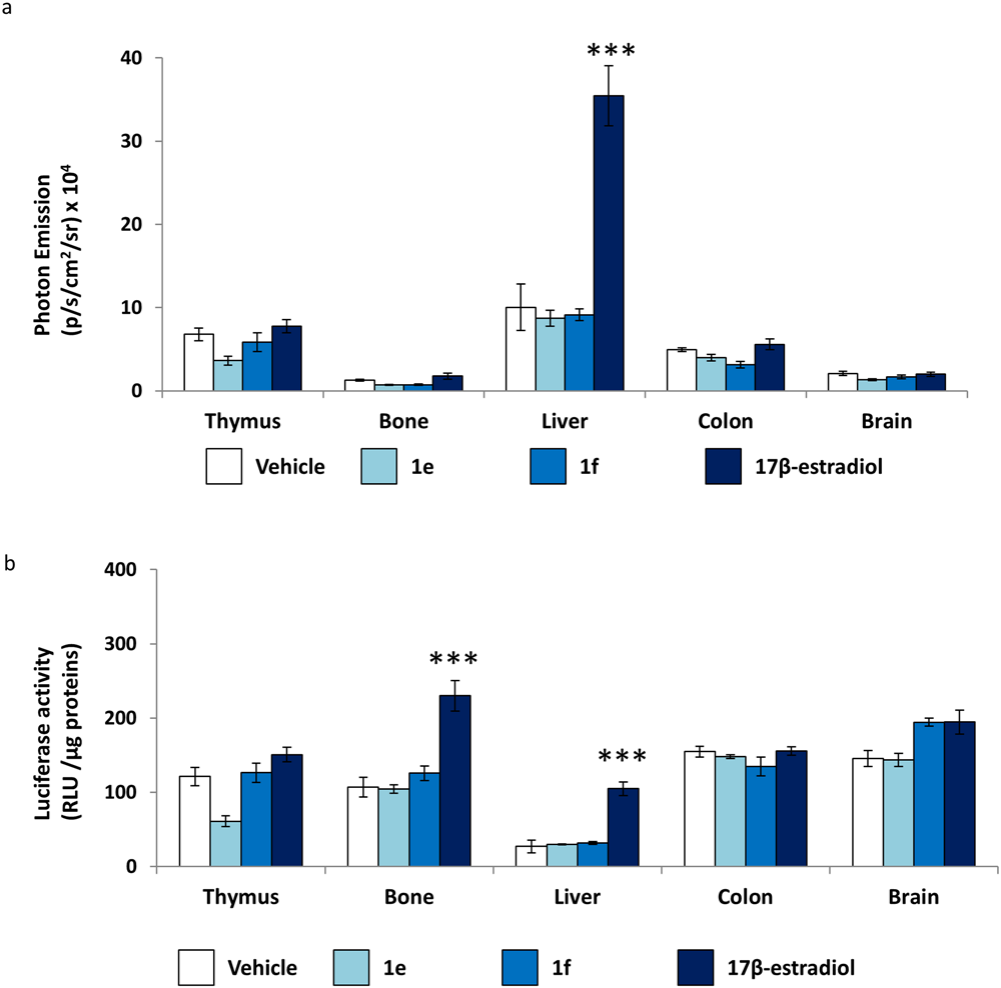
Analysis of the systemic effect of 1e and **1f** compounds to female ERE-*Luc* ovx mice. (**a**) *Ex vivo* BLI: photon emission measured in each organ as indicated. (**b**) Enzymatic Luciferse assay: luciferase enzymatic assay performed in indicated tissue. Bars represent the mean +/− SEM of photon emission measured in 4 animals/group. **p < 0.01 vs vehicle ***p < 0.001 vs vehicle ANOVA followed by Bonferroni’s test for multiple comparisons.

We next examined photon emission in a series of organs excised from the mice euthanized at 6 hours after treatment by measuring photon emission with the CCD camera (Fig. 6a) and by the more sensitive enzymatic assay in tissue extracts of each organ (Fig. 6b). This latter experiment clearly demonstrated that, contrary to 17β-estradiol, the two NCE did not induce bioluminescence in any of the organs investigated and further indicated that **1e** and **1f** were devoid of any systemic activity.

**Figure 7:**
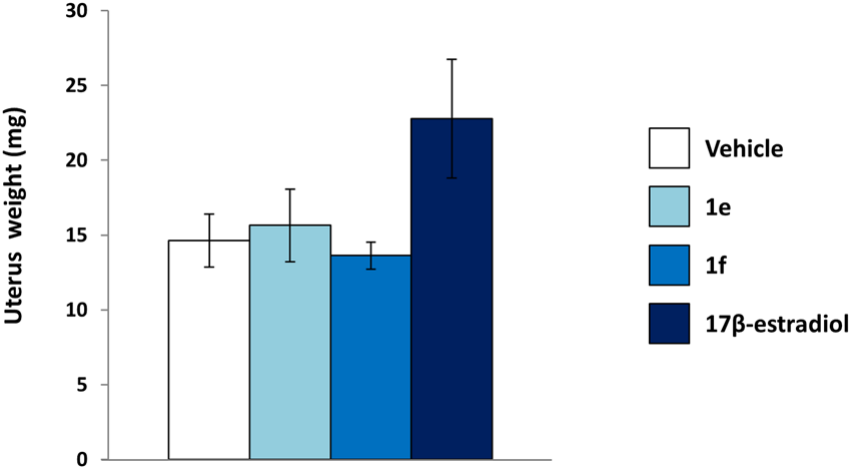
Uterus weight of ERE-*Luc* mice treated with synthetic and natural estrogens. Analysis of average weight of uterus of ERE-*Luc* mice after 6 hours of indicated treatment. Bars represent the mean ± SEM of uterus weigh measured in 4 animals/group.

The final study aiming at investigating the systemic effect of the NCE was done by measuring the uterus weight of all treated animal as the golden standard for the quantitative measure of estrogenic compounds. Figure 7 shows that, 6 h after treatment, with **1e** and **1f** the weight of the uterus was indistinguishable from controls; conversely, the average uterus weight of the mice treated with 17β-estradiol was 22.8 mg, *i*.*e* 35% higher than the average weight of controls (n = 4).

#### Proliferative potential and healing efficiency of **1e** and **1f** when administered

Wound healing is a dynamic process consisting of four continuous, overlapping, and precisely programmed phases: hemostasis, inflammation, proliferation and tissue growth (proliferation) and remodeling (maturation). The healing effect of estrogens may be exerted at several levels, certainly the ability of the hormone to accelerate macrophage polarization towards the M2-reparative polarization stage plays a relevant role, however estrogens may also facilitate local cell proliferation and the formation of the granulation tissue, epithelialization, and wound contraction. We therefore planned to carry out experiments with the MITO-*Luc* ^19^ a transgenic mouse carrying a reporter of mitosis, aimed at demonstrating the local and systemic proliferative potential of the synthetic estrogens we generated.

**Figure 8:**
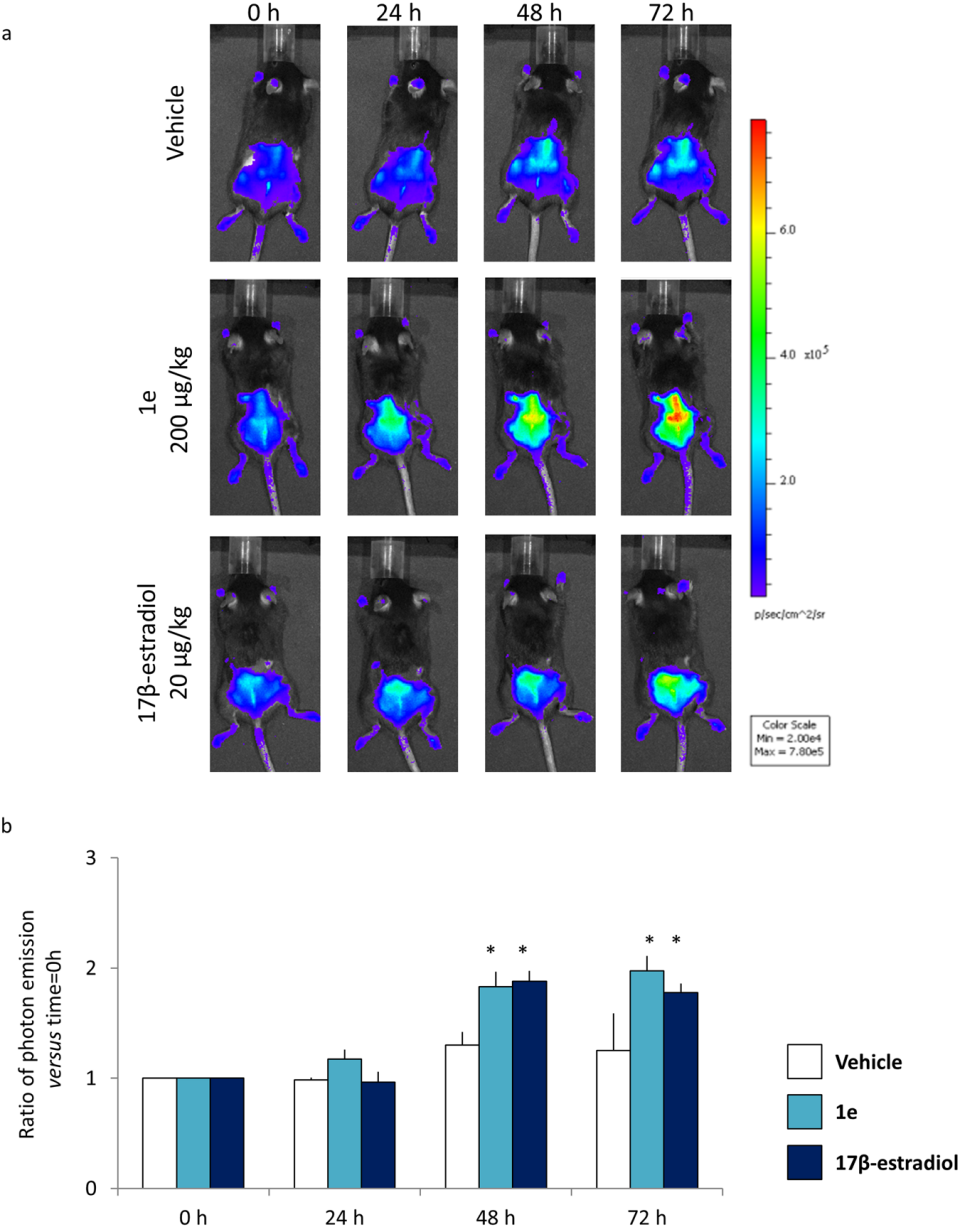
Proliferation analysis. (**a**) *In vivo* wound healing bioluminescence imaging of MITO-*Luc* after indicated treatments. Photos portray whole body (dorsal view) at 0, 24, 48, 72 hours after treatments, in a pseudocolor image processed to indicate the intensity of photon emission in wound areas. (**b**) *In vivo* BLI: quantification of photon emission in the wound area at the indicated time point. Each bar represents the mean ratio versus time = 0 h +/− SEM of photon emission measured in 4 animals/group. *p < 0.001 vs time 0, ANOVA followed by Bonferroni’s test for multiple comparisons.

The experiments were done with the **1e** compound as completely deprived of any activity on the uterus (Fig. 6). Ovariectomized MITO-*Luc* mice (n = 4 each group) were injected with vehicle (corn oil), 200 µg/kg **1e** and 20 µg/kg 17β-estradiol subcutaneously in the area surrounding the wound. Photon emission was measured in the wound area at the following time: 0, 24, 48 and 72 hours post-treatment. Figure 8 shows that, 48 hours after administration of **1e** and 17β-estradiol, photon emission from the wound area was significantly increased; the maximal activity of both treatments with **1e** and 17β-estradiol was observed at h 72 (87% increase of photon emission from wound of animals treated with 200 µg/kg **1e** and 78% increase in 20 µg/kg 17β-estradiol treated animals). In animals treated with the vehicle photon emission was also increased, but at much lower extent (+13% versus time 0), indicating that both **1e** and 17β-estradiol were able to regulate the extent of proliferation in the area surrounding the wound (Fig. 8).

**Figure 9:**
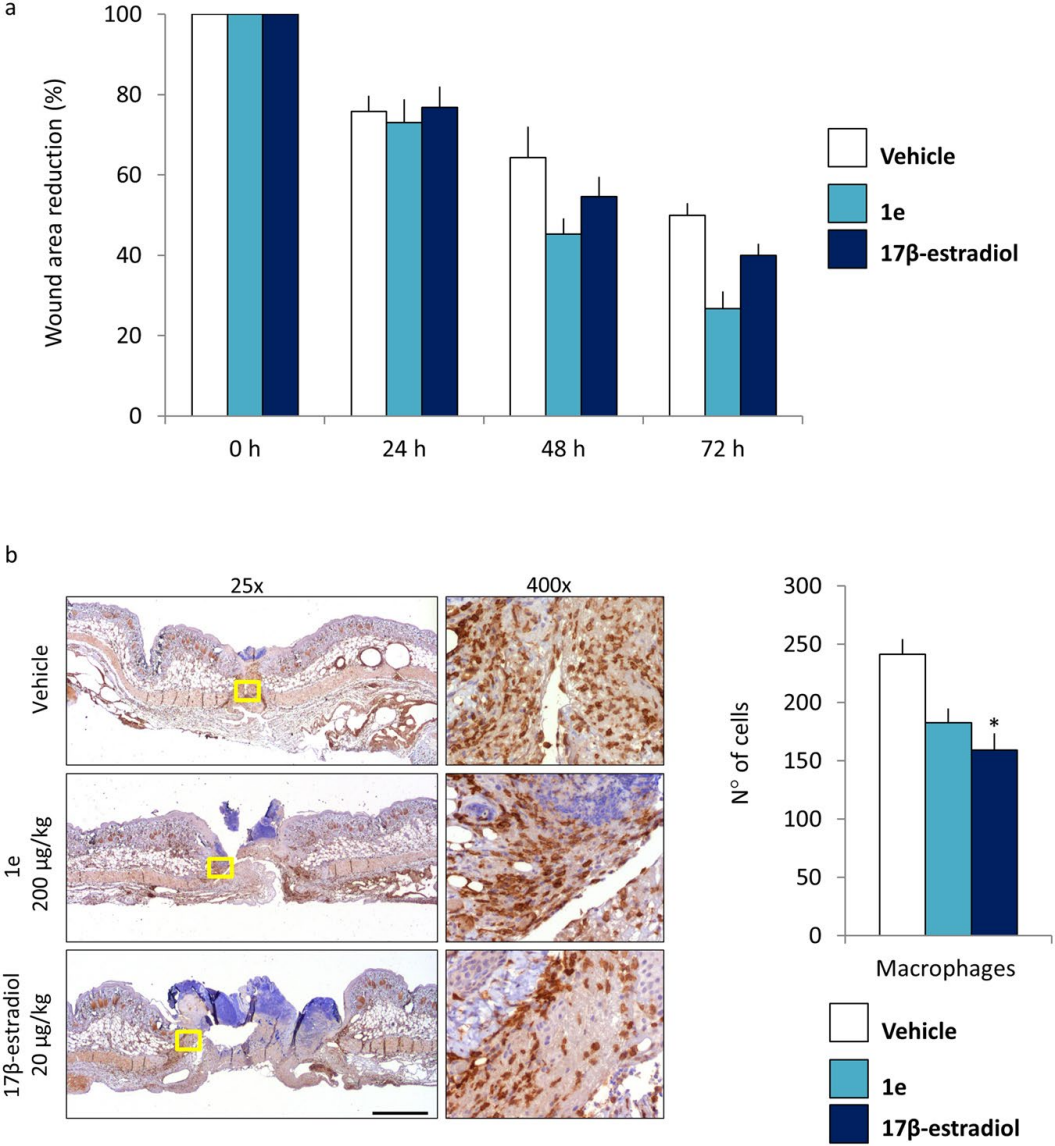
Reduction of the wound areas after treatment with 1e and 17β-estradiol. (**a**) Wound area measurement: each bar represents the mean ratio versus time = 0 h +/− SEM expressed as percentage of wound area measured in 4 animals/group. (**b**) Representative staining of wound explants at 72 hours after treatments. Staining was done with Iba1 antibody. Pictures were taken at 25x and 400x. Bar = 2 mm. The number of macrophages cells around wound site: each bar represents the mean +/−SEM, macrophages (Iba1 positive cells) were counted by analyzing n = 6 fields (vehicle), n = 11 fields (**1e**) and n = 10 fields (17β-estradiol) at 400x enlargement. (*p < 0.1 vs vehicle, ANOVA followed by Bonferroni’s test for multiple comparisons).

To investigate to which extent the increased proliferation observed after the pharmacological treatments was functional to the healing process, we measured the size of the wound (Fig. 9a). 72 hours after the treatments we observed a 73% wound area reduction in **1e** treated animals, 60% in 17β-estradiol treated animals and 50% in vehicle animals. These data suggested that **1e** accelerates wound healing.

To better understand whether the healing effect of **1e** could be attributed to its anti-inflammatory action, at the end of *in vivo* imaging (72 hours after treatments) the tissue surrounding the wound was dissected and subjected immunohistochemical analysis using the pan macrophage marker Iba1. As shown in Fig. 9b, in the animals treated with **1e** and 17β-estradiol the number of macrophages in the wound area significantly lower than in controls (25% and of 44% respectively). This observation confirmed that the estrogenic compounds were able to regulate the immune response at local level.

**Figure 10:**
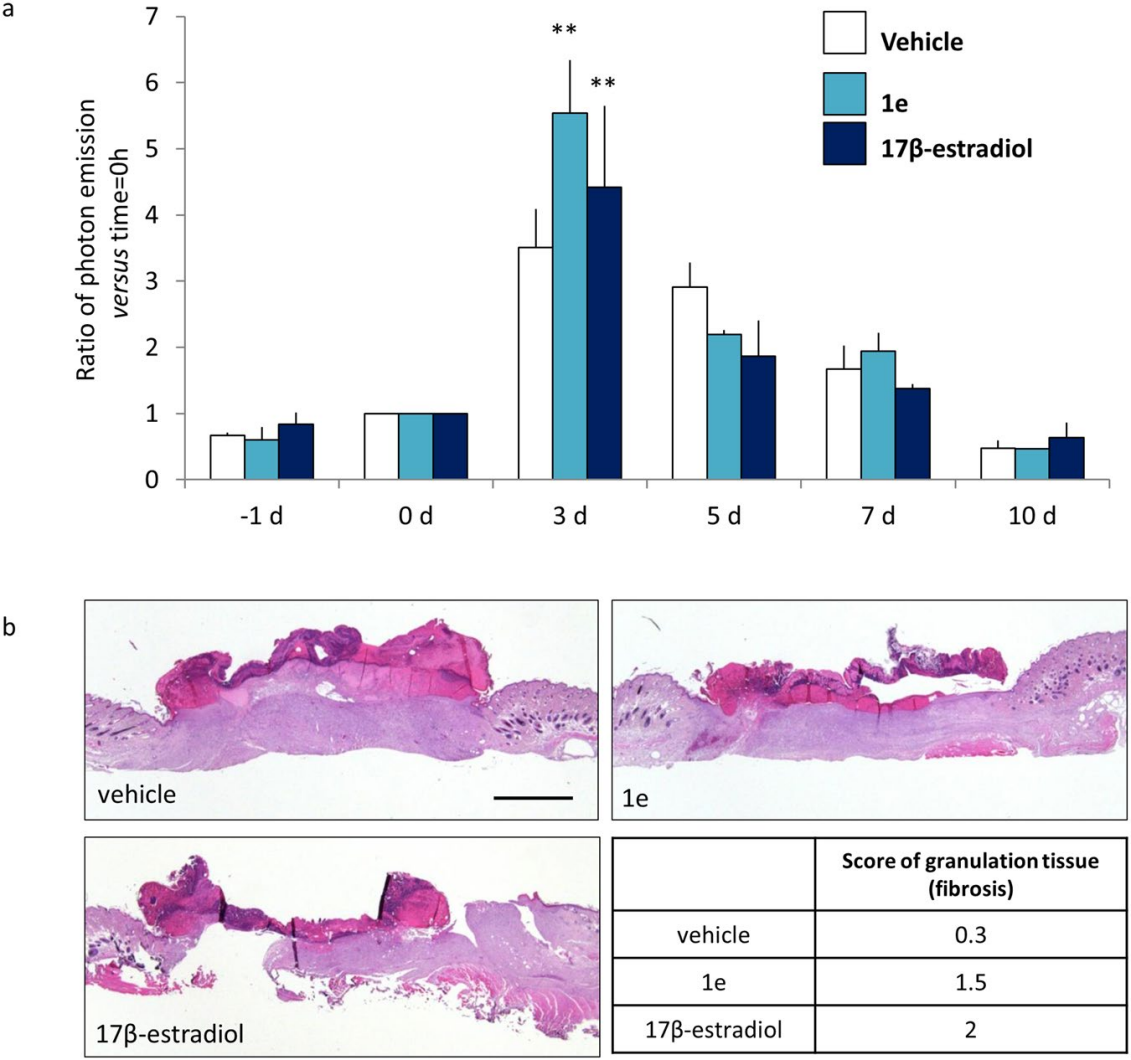
**1e** compound and 17β- estradiol accelerate wound healing. (**a**) *In vivo* BLI: round wound area photon emission quantification at indicated time point in MITO-*Luc* mice. Each bar represents the mean ratio versus time = 0 h +/− SEM of photon emission measured in 2 animals/group. (**p < 0.01 vs 0 h, ANOVA followed by Bonferroni’s test for multiple comparisons). (**b**) Hematoxylin-eosin representative staining of wound section at 10 days after the indicated treatments. The histoscore value for granulation tissue (fibrosis) is shown as mean, each histoscore consists of 4 random fields/groups, the criteria for the assignation of histoscore value are: 1 = wound bed partially covered with granulation tissue; 2 = thin granulation over the whole wound bed; 3 = thick granulation over the whole wound bed. Bar = 2 mm.

Next, we investigated the effects of **1e** with respect to 17β-estradiol using ovx MITO-*Luc* mice at longer time. The first day of the experiment, one round full thickness wound was made on each mouse (n = 2) and then all mice were treated with vehicle, 200 µg/kg **1e** or 20 µg/kg 17β-estradiol by subcutaneous injection around the round wound. The measure of photon emission from the wound area was done for several days after the wounding. 3 days after treatment the bioluminescent signal is highest (Fig. 10 Panel a). The intensity of photon emission was significantly decreased 2 days later and kept decreasing to reach, at day 10, values very comparable to those measured before the wounding (time = −1 day). The histological analyses done on the explanted wound tissues at day 10 showed that in the animals treated with **1e** there was a significantly higher granulation tissue than (histoscore mean = 1.5) in those injected with vehicle (histoscore mean = 0.3) (Fig. 10 panel b).

#### Comparative analysis of **1e** and 17β-estradiol systemic proliferative activity

**Figure 11:**
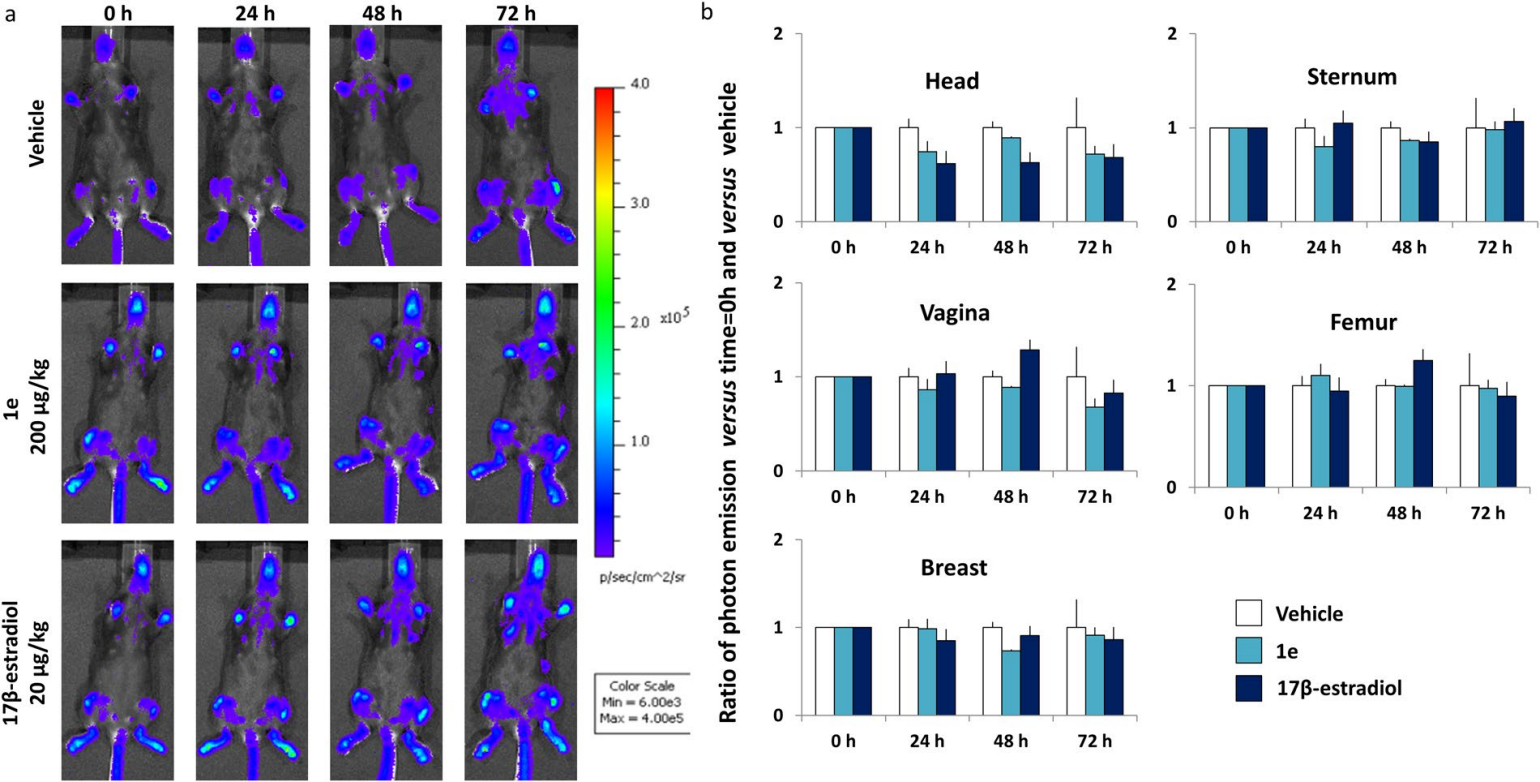
**1e** compound does not induce proliferation at systemic level in the MITO-*Luc* mouse. (**a**) *In vivo* whole body bioluminescence imaging of MITO-*Luc* after indicated treatments. Photos portray whole body (ventral view) at 0, 24, 48, 72 hours after treatments in a pseudocolor image processed to indicating the intensity of photon emission in whole body. (**b**) *In vivo* BLI: photon emission from selected body areas were measured. Bars represent the mean ratio versus t = 0 h and vs vehicle ± SEM of photon emission measured in 4 animals/group.

The study was done in the MITO-*Luc* mice. Photon emission was measured in the following body areas: head, sternum, vagina, femur and breast (ventral view, as indicated in Fig. 11a) at 0, 24, 48 and 72 hours after the local, s. c., injection of vehicle, 200 µg/kg 1e and 20 µg/kg 17β-estradiol (n = 4). No increase of bioluminescence was measured in **1e** treated animals respect to vehicle at any time point (Fig. 11b).

This was not the case with the animals treated with 17β-estradiol where 48 hours after the local s.c. injection of 17β-estradiol we observed that the bioluminescent signal showed a trend to increase, likely due to the uterotrophic effect of the hormone. This observation further underlines the lack of systemic effects of the **1e** compound.

## Conclusions

In the present study we have prepared a family of compounds active through the ER characterized by a structure that should ensure their rapid metabolism and limited systemic action when administered *in vivo*. Most of compounds tested, and in particular **1e** and **1f**, showed a significant affinity for ERs and were able to efficiently induce ER transcriptional activity.

The biological activity of **1e** tested in a well-characterized wound healing murine model^8^ showed a significant effect on wound healing and inflammatory processes. **1e** proved to be very effective in accelerating healing. Several studies suggest that the beneficial effects of estrogens on the healing process is in the dampening of the inflammatory response, in particular it was reported that accelerated healing was correlated with a reduction in infiltrating macrophages numbers^1, 3, 4^. The current study supports this view by showing a decreased number of Iba1 positive macrophages in the granulation tissue (Fig. 9b); the increase of proliferation in the **1e** and 17β-estradiol treated animals may be in line with the view of an accelerated transition from inflammatory phase to proliferative phase. In this regard, **1e** showed an activity comparable to that one of the natural estrogen. Most importantly, **1e** administered s.c. at the dose of 200 μg/kg in the wound area proved to be devoid of systemic effects as indicated by the lack of ER transcriptional activity in the ERE-*Luc* model, of bioluminescence signaling in the MITO-*Luc mouse* and the lack of uterotrophic effect. All parameters were clearly affected by 17β-estradiol (Figs 5, 6 and 11).

Our study, by identifying a new class of molecules locally active through the ERs in the wound healing process confirms the results of our previous work and add new avenues in the search of novel drugs for a still unmet medical need.

## Methods

### Materials and Methods

All solvents were analytical grade. TLC: Merck silica gel 60 F254. CC: silica gel 60, 70–230 mesh ASTM. IR spectra: Shimadzu-FTIR 8400S infrared spectrophotometer, in cm-1. ^1^H and ^13^C NMR: Bruker AC 300P operating at 300.13 and 75 MHz respectively; compound purity was evaluated by HPLC analysis: Shimadzu LC-10AD; RID detector; column, Macherey-Nagel 250/4 Nucleosil 100-5; flow, 0.8 mL/min; T, 35 °C; all solvents were HPLC grade; t_R_ in min. Mp: Mettler-FP-61 apparatus (uncorrected). HR mass spectra were recorded with a Micromass Q-TOF micro mass spectrometer (Waters). FIA-MS experiments were performed on a linear ion trap mass spectrometer (LXQ Thermo Finningan) equipped with a heated electrospray ionization (HESI) source. Operating conditions of the HESI were as follows: source temperature: 50 °C; ion spray voltage = +4.0 kV; sheath gas = 5 (arbitrary scale); capillary temperature = 275 °C. Methanolic solutions of samples (1 × 10^−5^ M) were infused via a syringe pump at a flow rate of 3–5 μL/min. Unless otherwise specified, all organic solutions were dried over anhydrous Na_2_SO_4_ and evaporated to dryness under reduced pressure.

### General procedure of the preparation of compounds 1

A mixture of **3** ^8^ (1.0 mmol) in CH_3_CN (40 mL), TEMPO (30 mg, 0.19 mmol) and sodium phosphate buffer (0.67 M, 6 mL, pH 6.7) was stirred at 40 °C. To this mixture, NaClO (5.25%, 0.24 mL, 0.20 mmol) and NaClO_2_ (80%, 0.34 g, 3.0 mmol) were added portionwise simultaneously.

The solution became slowly brownish and was left under stirring at 40 °C till disappearance of the starting material (generally 2–4 hours, TLC: EtOAc). To the reaction mixture H_2_O (40 mL) was added and pH adjusted to 8 with 4N NaOH. The reaction flask was then cooled to 0 °C and quenched with a Na_2_SO_3_ aqueous solution (2 g in 30 mL). The whole was then extracted with methyl-*t*-butylether and the organic phase discarded. After adjusting the pH to 4 the aqueous phase was thoroughly extracted with EtOAc. Combined organic phases were washed with water, brine, dried over anhydrous Na_2_SO_4_ and evaporated to dryness to give carboxylic acids **4**. The latter was used as crude in the next reaction. Thus to an ice-bath cooled solution of **4** and DMAP (30 mg, 0.25 mmol) in the proper alcohol (20 mL), EDC (0.23 g, 1.2 mmol) was slowly added in 40 minutes. The ice bath was removed and the whole allowed to stir at rt till disappearance of the starting material (generally 20 hours, TLC: EtOAc/hexane = 6/4). The mixture was then concentrated under reduced pressure, H_2_O was added and the whole extracted with EtOAc. The combined organic phases were washed with NaHCO_3_ s.s., brine, dried over anhydrous Na_2_SO_4_ and evaporated to dryness to give crude **5**.

The crude product of the previous reaction was dissolved in MeOH (20 mL) and a 0.1 M *p*-toluensulphonic acid solution was added to adjust the pH to 2. The resulting solution was left under stirring at rt until TLC showed the complete disappearance of the starting material (generally 1 h). The solution was then diluted with EtOAc (80 mL) and washed to neutrality with a saturated NaHCO_3_ aqueous solution, and the aqueous phase was extracted with EtOAc. The combined organic phases were collected, washed with brine and dried. After filtration, evaporation of the solvent and purification of the crude product, gave **1** as a pale yellow viscous oil.

### 3,17β-Hydroxy-17α-(4′carbomethoxy-1′-butyn-1′-yl)estra-1,3,5-(10)-triene (1a)

Yield: 70%

CC: EtOAc/*n*-hexane, from 2/8 to 3/7

^1^H NMR(CDCl_3_): 7.15 (d, *J* = 8.4 Hz, 1 H), 6.63 (dd, *J*_1_ = 2.7, *J*_2_ = 8.4 Hz, 1 H), 6.57 (d, *J* = 2.7 Hz, 1H), 3.69 (s, 1H), 2.84–2.76 (m, 2H), 2.57 (s, 3H), 2.38–2.12 (m, 4H), 2.03–1.22 (m, 12H), 0.86 (s, 3H).

^13^C NMR(CDCl_3_): 172.6, 153.5, 138.4, 132.7, 126.7, 115.4, 112.8, 84.7, 84.4, 80.0, 52.0, 49.6, 47.0, 43.7, 39.6, 39.1, 33.8, 33.0, 29.8, 27.3, 26.6, 22.9, 15.0, 12.9.

IR (CHCl_3_): 3599, 2933, 1734.

HPLC: EtOAc/*n*-hexane = 27/73; tR = 19.2 min, purity = 97.8%.

HRMS(ESI): Calc for C_24_H_30_O_4_ 405.2042 (M + Na)+; found 405.2053.

### 3,17β-Hydroxy-17α-(4′carbethoxy-1′-butyn-1′-yl)estra-1,3,5-(10)-triene (1b)

Yield: 65%

CC: EtOAc/*n*-hexane = 2/8

^1^H NMR(CDCl_3_): 7.13 (d, *J* = 8.4 Hz, 1 H), 6.65 (dd, *J*_1_ = 2.5 Hz, *J*_2_ = 8.3 Hz, 1H), 6.58 (d, *J* = 2.3 Hz, 1H), 4.16 (q, *J* = 7.1, 2H), 2.88–2–73 (m, 2H), 2.62–2.51 (m, 4H), 2.40–2.10 (m, 3H), 2.09–1.91 (m, 2H), 1.90–1.55 (m, 5H), 1.53–1.22 (m, 7H), 0.84 (s, 3H).

^13^C NMR(CDCl_3_): 172.5, 153.7, 138.2, 132.3, 126.5, 115.4, 112.9, 84.6, 84.3, 80.1, 61.0, 49.5, 47.3, 43.7, 39.6, 39.0, 34.0, 32.9, 29.7, 27.3, 26.5, 22.9, 14.9, 14.3, 12.9.

IR(CHCl_3_): 3595, 2360, 2343, 1732.

HPLC: EtOAc/*n*-hexane = 30/70; tR = 8.8 min, purity = 94.8%.

HRMS(ESI): Calc for C_25_H_32_O_4_ 419.2198 (M + Na)+; found 419.2219.

### 3,17β-Hydroxy-17α-(4′carbopropoxy-1′-butyn-1′-yl)estra-1,3,5-(10)-triene (1c)

Yield: 61%

CC: EtOAc/n-hexane = 2/8

^1^H NMR (CDCl_3_): 7.15 (d, *J* = 8.3 Hz, 1H), 6.64 (dd, *J*_1_ = 2.7 Hz, *J*_2_ = 8.4 Hz, 1H), 6.57 (d, *J* = 2.6 Hz, 1H), 4.05 (t, *J* = 6.7 Hz, 2H), 2.87–2.76 (m, 2H), 2.62–2.52 (m, 4H), 2.37–2.11 (m, 3H), 2.07–1.92 (m, 2H), 1.90–1.56 (m, 7H), 1.53–1.21 (m, 4 H), 0.93 (t, J = 7.4 Hz, 3H), 0.85 (s, 3H).

^13^C NMR (CDCl_3_): 172.4, 153.5, 138.4, 132.7, 126.7, 115.4, 112.8, 84.6, 84.5, 80.1, 66.5, 49.5, 47.3, 43.7, 39.5, 39.1, 34.0, 33.0, 29.8, 27.3, 26.6, 22.9, 22.1, 15.0, 12.9, 10.5.

IR (CHCl_3_): 3390, 2330, 1728.

HPLC: EtOAc/*n*-hexane = 30/70; tR = 7.6 min, purity = 91.2%.

HRMS(ESI): Calc for C_26_H_34_O_4_ 433.2355 (M + Na)+; found 433.2371.

### 3,17β-Hydroxy-17α-(4′carbo-iso-propoxy-1′-butyn-1′-yl)estra-1,3,5-(10)-triene (1d)

Yield: 45%

CC: EtOAc/*n*-hexane = 2/8

^1^H NMR (CDCl_3_): 7.14 (d, *J* = 8.4 Hz, 1H), 6.64 (dd, *J*_1_ = 2.7 Hz, *J*_2_ = 8.4 Hz, 1H), 6.57 (d, *J* = 2.6 Hz, 1H), 5.03(spt, *J* = 6.2 Hz, 1H), 2.85–2.75 (m, 2H), 2.63–2.44 (m, 4H), 2.36–1.10 (m, 20H), 0.86 (s, 3H).

^13^C NMR (CDCl_3_): 171.9, 153.6, 138.3, 132.6, 126.6, 115.4, 112.8, 84.6, 84.5, 80.1, 68.4, 49.5, 47.3, 43.7, 39.6, 39.1, 35.0, 33.0, 29.8, 27.3, 26.6, 22.2, 21.9, 15.0, 12.9.

IR (CHCl_3_): 3414, 1724.

HPLC: EtOAc/*n*-hexane = 27/73; tR = 8.9 min, purity = 99.7%

HRMS(ESI): Calc for C_26_H_34_O_4_ 433.2355 (M + Na)+; found 433.2364.

### 3,17β-Hydroxy-17α-(4′carbobutoxy-1′-butyn-1′-yl)estra-1,3,5-(10)-triene (1e)

Yield: 65%

CC: EtOAc/*n*-hexane = 2/8

^1^H NMR (CDCl_3_): 7.13 (d, *J* = 8.4 Hz, 1H), 6.65 (dd, J_1_ = 2.5 Hz, *J*_2_ = 8.4 Hz, 1H), 6.58 (d, *J* = 2.4 Hz, 1H), 4.10 (t, J = 6.6 Hz, 2H), 2.88–2.73 (m, 2H), 2.65–2.49 (m, 4H), 2.37–2.10 (m, 3H), 2.09–1.92 (m, 2H), 1.90–1.54 (m, 7H), 1.53–1.22 (m, 6H), 0.92 (t, J = 7.3 Hz, 3H), 0.86 (s, 3H).

^13^C NMR (CDCl_3_): 172.5, 153.7, 138.3, 132.4, 126.6, 115.4, 112.8, 84.6, 84.4, 80.1, 64.9, 49.5, 47.3, 43.7, 39.5, 39.0, 34.0, 33.0, 30.7, 29.8, 27.3, 26.6, 22.9, 19.2, 15.0, 13.8, 12.9.

IR (CHCl_3_): 3597, 1732.

HPLC: EtOAc/*n*-hexane = 30/70; tR = 6.9 min, purity = 94.4%.

HRMS(ESI): Calc for C_27_H_36_O_4_ 447.2511 (M + Na)+; found 447.2520.

### 3,17β-Hydroxy-17α-(4′carbamyloxy-1′-butyn-1′-yl)estra-1,3,5-(10)-triene (**1f**)

Yield: 50%

CC: EtOAc/*n*-hexane = 2/8

^1^H NMR (CDCl_3_): 7.12 (d, *J* = 8.4 Hz, 1H), 6.67 (dd, *J*_1_ = 1.9 Hz, *J*_2_ = 8.4 Hz, 1H), 6.60 (d, *J* = 1.9 Hz, 1H), 4.10 (t, *J* = 6.8 Hz, 2H), 2.87–2.68 (m, 2H), 2.57 (s, 4H), 2.37–1.24 (m, 19H), 0.92–0.87 (m, 6H).

^13^C NMR (CDCl_3_): 172.7, 153.8, 138.0, 132.1, 126.4, 115.4, 112.8, 84.6, 84.2, 80.0, 65.2, 49.4, 47.2, 43.6, 39.5, 38.9, 33.9, 32.9, 29.7, 28.3, 28.0, 27.3, 26.5, 22.8, 22.3, 14.9, 14.0, 12.9.

IR (CHCl_3_): 3597, 2933, 1732.

HPLC: EtOAc/*n*-hexane = 30/70; tR = 6.6 min, purity = 94%.

HRMS(ESI): Calc for C_28_H_38_O_4_ 461.2668 (M + Na)+; found 461.2655.

### 3,17β-Hydroxy-17α-(5′carbomethoxy-1′-butyn-1′-yl)estra-1,3,5-(10)-triene (1g)

Yield: 51%

CC: EtOAc/*n*-hexane from 2/8 to 4/6

^1^H NMR (CDCl_3_): 7.16 (d, *J* = 8.5 Hz, 1H), 6.63 (dd, *J*_1_ = 8.4 Hz, *J*_2_ = 2.7 Hz, 1H), 6.56 (d, *J* = 2.7, 1H), 3.67 (s, 3H), 2.90–2.70 (s, 2H), 2.51–1.30 (m, 19H), 0.86 (s, 3H).

^13^C NMR (75.47 MHz, CDCl_3_): δ = 173.9, 153.5, 138.4, 132.8, 126.7, 115.4, 112.8, 85.171, 84.8, 80.2, 51.8, 49.7, 47.3, 43.8, 39.6, 39.3, 33.1, 29.8, 23.8, 26.6, 24.1, 22.9, 18.5, 13.0.

IR (CHCl_3_): 3599, 2933, 1734.

HPLC: EtOAc/*n*-hexane = 27/73; tR = 17.3 min, purity = 94.2%.

HRMS(ESI): Calc for C_25_H_32_O_4_ 419.2198 (M + Na)+; found 419.2180.

### 3,17β-Hydroxy-17α-(5′carbethoxy-1′-butyn-1′-yl)estra-1,3,5-(10)-triene (1h)

Yield: 51%

CC: EtOAc/*n*-hexane = 2/8

^1^H NMR (CDCl_3_): 7.17 (d, *J* = 8.4 Hz, 1H), 6.63 (dd, *J*_1_ = 2.1 Hz, *J*_2_ = 8.4 Hz, 1H), 6.56 (s,1H), 4.13 (q, J = 7.2 Hz, 2H), 2.89–2.68 (m, 2H), 2.47–1.15 (m, 22H), 0.87 (s, 3H).

^13^C NMR (CDCl_3_): 173.5, 153.6, 138.4, 132.7, 126.7, 115.4, 112.8, 85.3, 84.8, 80.2, 60.6, 49.6, 47.3, 43.8, 39.6, 39.2, 33.4, 33.1, 29.8, 27.4, 26.6, 24.1, 22.9, 18.4, 14.3, 13.0.

IR (CHCl_3_): 3597, 3391, 2937, 1728.

HPLC: EtOAc/*n*-hexane = 27/73; tR = 13.8 min, purity = 99.9%.

HRMS(ESI): Calc for C_26_H_34_O_4_ 433.2355 (M + Na)+; found 433.2340.

### 3,17β-Hydroxy-17α-(4′carboxy-1′-butyn-1′-yl)estra-1,3,5-(10)-triene (7)

To a rt stirred solution of **1b** (0.10 mmol) in EtOH (12 mL) a 0.1 M KOH solution (10 mL) was added. The whole was allowed to stir for 2 h when TLC monitoring (EtOAc/hexane = 6/4) showed the reaction completion. The reaction mixture was then acidified with 0.1 M HCl and thoroughly extracted with EtOAc. Combined organic extracts were washed with brine, dried and evaporated to dryness to give crude **7** which was purified by CC (EtOAc, 90% yield).

^1^H NMR (CD_3_COCD_3_): 7.09 (d, *J* = 8.4 Hz, 1H), 6.60 (dd, *J*_1_ = 2.4 Hz, *J*_2_ = 8.4 Hz, 1H), 6.52 (d, *J*_1_ = 2.4 Hz,1H), 2.85–2.66 (m, 2H), 2.55–2.44 (m, 4H), 2.37–2.26 (m, 1H), 2.22–2.08 (m, 2H), 2.03–1.60 (m, 6H), 1.47–1.22 (m, 4H), 0.86 (s, 3H).

^13^C NMR (CD_3_COCD_3_): 173.3, 155.8, 138.4, 132.1, 127.1, 115.9, 113.6, 86.0, 84.1, 79.7, 50.1, 48.0, 44.5, 40.6, 40.0, 34.1, 33.7, 30.3, 28.1, 27.4, 23.5, 15.3, 13.3.

IR(ATR): 3234, 1701.

HPLC: Chiralcel OB; *i*-PrOH/*n*-hexane = 15/85 + 0.1% TFA; tR = 8.1 min, purity = 98%, flow; 1 mL/min.

HRMS(ESI): Calc for C_23_H_28_O_4_ 391.1880 (M + Na)+; found 391.1874.

### General preparation of compounds 6

To a stirred solution of **3** (1.0 mmol) in 12 mL of MeOH a 0.1 M *p*-toluensulphonic acid solution was added to adjust the pH to 2. The resulting solution was left under stirring at rt. After complete disappearance of the starting material (generally 1 h) the solution was diluted with EtOAc (20 mL), washed to neutrality with a saturated NaHCO_3_ aqueous solution, and the aqueous phase extracted with EtOAc. The combined organic phases were collected, washed with brine and dried. After filtration, evaporation of the solvent gave crude **6**, which were purified by CC.

### 3,17β-Hydroxy-17α-(4′hydroxy-1′-butyn-1′-yl)estra-1,3,5-(10)-triene (6a)

Yield: 93%

CC: EtOAc/*n*-hexane 5/5

White powder

^1^H NMR (CD_3_OD): 7.10 (d, *J* = 8.5 Hz, 1H), 6.56 (dd, *J*_1_ = 8.4 Hz, *J*_2_ = 2.7 Hz, 1H), 6.49 (d, *J* = 2.6 Hz, 1H), 3.69 (t, *J* = 6.4 Hz, 2H), 2.87–2.72 (m, 2H), 2.36 (t, *J* = 7.0 Hz, 3H), 2.26–2.10 (m, 2H), 2.02–2.85 (m, 3H), 1.80–1.69 (m, 5H), 1.50–1.28 (m, 4H), 0.87 (s, 3H).

^13^C NMR (CD_3_OD): 155.9, 138.8, 132.6, 127.2, 116.0, 113.7, 85.8, 85.4, 80.6, 61.7, 50.8, 48.4, 45.2, 41.2, 40.0, 34.2, 32.9, 30.8, 28.7, 27.8, 23.7, 16.0, 13.5.

IR(ATR): 3404, 3182.

HPLC: EtOAc/*n*-hexane = 50/50; tR = 15.3 min, purity = 99.9%.

HRMS(ESI): Calc for C_23_H_30_O_3_ 377.2087 (M + Na)+; found 377.2078

Mp (EtOAc/*n*-hexane) = 151.6–153.3 °C.

### 3,17β-Hydroxy-17α-(5′hydroxy-1′-butyn-1′-yl)estra-1,3,5-(10)-triene (6b)

Yield: 95%

CC: EtOAc/*n*-hexane from 5/5 to 6/4

White powder

^1^H NMR (CD_3_OD): 7.07 (d, *J* = 8.5 Hz, 1H), 6.53 (dd, *J* = 8.4, 2.5 Hz, 1H), 6.47 (d, *J* = 2.4 Hz, 1H), 3.57 (t, *J* = 6.1 MHz, 2H), 2.80–2.69 (m, 2H), 2.32–1.21 (m, 19H), 0.84 (s, 3H).

^13^C NMR (CD_3_OD): 155.8, 138.8, 132.6, 127.2, 116.0, 113.7, 86.3, 85.4, 80.5, 62.4, 50.8, 48.4, 45.2, 41.1, 40.0, 34.2, 32.8, 30.7, 28.6, 27.7, 26.4, 23.7, 19.3, 13.5.

IR(ATR): 3373, 3227.

HPLC: EtOAc/*n*-hexane = 50/50; tR = 14.3 min, purity = 97.6%

HRMS(ESI): Calc for C_24_H_32_O_3_ 391.2244 (M + Na)+; found 391.2241.

Mp (EtOAc/*n*-hexane) = 141.4–142.1 °C.

### Cell Culture

The study was carried out using B17 clone of MCF-7 cells previously generated in our laboratory by stable transfection of a plasmid containing the luciferase gene under the control of an estrogen responsive promoter 10. Cells were grown in RPMI 1640 supplemented with 10% FBS (Euroclone, U.K.), 50 U/mL penicillin G, 50 µg/mL streptomicyn sulphate, 1 mM sodium pyruvate, at 37 °C at 99% humidity and 5% CO_2_. Cells were split twice a week by seeding 2 × 106 cells in 100 mm diameter Petri (Corning) dishes.

### *In vitro* study of agonistic activity

For the *in vitro* measurement of activity of the new chemical entities, 1 × 105 cells were seeded in twenty-four well plate in phenol red-free RPMI 1640 medium (Invitrogen) supplemented with 10% dextran-coated charcoal stripped-FBS, 1% of non-essential amino acid, 50 U/mL penicillin G, 50 µg/mL streptomicyn sulphate, 1 mM sodium pyruvate, 2 mM L-Glutamine and kept at 37 °C in a humidified incubator for 24 h. Next, culture medium was replacing with phenol red-free RPMI 1640 supplemented with 1% stripped-FBS for a minimum of 2 h before adding 17β-estradiol or new compounds at doses indicated in a Fig. 3. After 24 h cells were rinsed once with PBS before preparing the protein extract for the determination of luciferase content. Each experiment was carried out in duplicate.

### Experimental animals

ERE-*Luc* and MITO-*Luc* mice were ovariectomized at two months of age; the experiment was done 2 weeks after surgery to allow the animals to recover and to decrease the levels of circulating estrogens. The animals were fed ad libitum with a special estrogen free diet (AIN 93 M, Mucedola) (Ciana *et al*. Endocrinology 2005) and housed in individually ventilated plastic cages within a temperature range of 22–25 °C, relative humidity of 50% ± 10% and under an automatic cycle of 12 h light/dark (lights on at 07:00 am). All animal experimentation was carried out in accordance with the ARRIVE and European Guidelines for Animal Care. All animal experiments were approved by the Italian Ministry of Research and University and controlled by a Departmental panel of experts. For the correct dosage of the compounds, the body weight of the animals (on empty stomach, fasted for at least 12–14 hours) was determined immediately before the treatment.

### Animals treatments

Ovariectomy was carried out as previously described^12^. Wound healing was done after shaving by VEET depilatory cream a day before the treatment. The 1 cm full-thickness incisional wound was made through the skin and panniculus carnosus muscle of ovariectomized MITO-*luc* using a surgical scalpel (blade n°10, carbon steel, Swann-Morton Ltd). Round full-thickness wounds were done by cutting out a section of full-thickness skin (around 9 mm in diameter) using fine scissors on the dorsal skin of the isoflurane-anesthetized animal. For *in vivo* treatment the compounds were administered onto the subcutaneous area surrounding the wound by multiple subcutaneous injection, immediately after the generation of the wound.

### *In vivo* imaging

For the semi-quantitative analysis of *in vivo* photon emission, animals were injected i.p. with 80 mg/kg of luciferin (Beetle Luciferin Potassium Salt; Promega, Madison, WI, USA) 15 min prior the imaging session. For the imaging, mice were anaesthetized using isofluorane (Isofluorane-Vet; Merial, Lyon, France) and kept under anesthesia during the 5 minutes of the session carried out with a CCD camera: both ventral and dorsal acquisition (Xenogen IVIS Lumina System; Caliper, PerkinElmer company). Photon emission in selected body areas was measured using the Living Image Software (Caliper, PerkinElmer company). The analysis was done in ventral view: whole body, thymus, hepatic area, abdomen, limbs, vagina and in dorsal view in wound area and expressed as photon/second/cm2/steradiant (p/s/cm2/sr).

### *Ex vivo* imaging

The selected organs were dissected from the animals who had received luciferine 20 min before euthanasia. The tissues were immediately subjected to *ex vivo* imaging session of 5 minutes. Photon emission was quantified with the Living Image Software (Caliper, PerkinElmer company) for each tissue and expressed as p/s/cm2/sr.

### Luciferase enzymatic assay

Luciferase enzymatic activity was measured in cells or selected tissue homogenates by a luminometer (Glomax™96 microplate luminometer; Promega) using Promega standard luciferin (Beetle Luciferin Potassium Salt) and expressed as Relative Light Units over 10 sec/μg protein (RLU/μg protein). Proteins concentrations were measured by Bradford.

### Histology, immunohistochemistry and image analysis

At the time of euthanasia, the wounds with surrounding healthy tissue were excised, distended on a strip of hard paper and fixed in 10% buffered formalin (pH = 6). Fixed samples were cut through the center of the wound and each half was embedded in paraffin “sideways on”. All fixed samples underwent routine processing in an automatic tissue processor and then paraffin embedding was carried out according to standard procedures. For histopathological examination 4 µm sections were routinely stained with Hematoxylin-Eosin (HE) and evaluated under a light microscope. The degree of re-epithelialization, the type and amount of granulation tissue and inflammation, which are the main features of wound healing, were scored separately. The scoring system is slightly modified compared to that applied by Tkalcević^20^.

In particular, the criteria for the assignation of granulations value are: 1 = wound bed partially covered with granulation tissue; 2 = thin granulation over the whole wound bed; 3 = thick granulation over the whole wound bed. Evaluation of macrophages was done through a specific antibody and digital image analysis: 4 µm sections from each wound healing sample were immunostained with primary rabbit monoclonal antibody against Iba1 antigen (Wako; #019-19741) to assess the number of macrophages.

The number of positive cells were counted using the ImageJ analysis program (http://rsb.info.nih.gov/ij/) in 400x microscopic fields selected at the periphery of the wound area.

### Statistical analysis

Analysis of transactivation data and calculation of EC50 values were performed by means of sigmoidal dose-response (variable slope) using PRISM5 software (GraphPad Software Inc.).

Analysis of statistical significance was done by ANOVA followed by Bonferroni’s test for multiple comparisons.

## Electronic supplementary material

Supplementary informations

## References

[1] Margolis DJ, Knauss J, Bilker W (2002). Hormone replacement therapy and prevention of pressure ulcers and venous leg ulcers. Lancet.

[2] Hardman MJ, Ashcroft GS (2008). Estrogen, not intrinsic aging, is the major regulator of delayed human wound healing in the elderly. Genome Biol..

[3] Ashcroft GS (1997). Estrogen accelerates cutaneous wound healing associated with an increase in TGF-beta1 levels. Nat. Med..

[4] Hardman MJ, Emmerson E, Campbell L, Ashcroft GS (2008). Selective estrogen receptor modulators accelerate cutaneous wound healing in ovariectomized female mice. Endocrinology.

[5] Emmerson E, Hardman MJ (2012). The role of estrogen deficiency in skin ageing and wound healing. Biogerontology.

[6] Thornton MJ (2013). Estrogens and Aging Skin. Dermatoendocrinol..

[7] Ghisletti S, Meda C, Maggi A, Vegeto E (2005). 17beta-estradiol inhibits inflammatory gene expression by controlling NF-kappaB intracellular localization. Mol. Cell. Biol..

[8] Villa A, Rizzi N, Vegeto E, Ciana P, Maggi A (2015). Estrogen accelerates the resolution of inflammation in macrophagic cells. Sci. Rep..

[9] Brufani M (2009). Novel locally active estrogens accelerate cutaneous wound healing. A preliminary study. Mol. Pharm..

[10] Laurent H, Gerhards E, Wiechert R (1975). New biologically active pregnan-21-oic acid esters. J. Steroid Biochem.

[11] Bucourt R, Vignau M, Torelli V (1978). New biospecific adsorbents for the purification of estradiol receptor. J. Biol. Chem..

[12] Labaree DC, Reynolds TY, Hochberg RB (2001). Estradiol-16-alphacarboxylic acid esters as locally active estrogens. J. Med. Chem..

[13] Gao H, Katzenellenbogen JA, Garg R, Hansch C (1999). Comparative QSAR analysis of estrogen receptor ligands. Chem. Rev..

[14] Zhao M (1999). Oxidation of primary alcohols to carboxylic acids with sodium chlorite catalyzed by TEMPO and bleach. J. Org. Chem..

[15] Madhup K, Dhaon MK, Olsen RK, Ramasamy K (1982). Esterification of N-protected Alpha-amino Acids with Alcohol/carbodiimide/4-(dimethylamino)pyridine. Racemization of aspartic and glutamic acid derivatives. J. Org. Chem..

[16] Ciana P (2001). Engineering of a mouse for the *in vivo* profiling of estrogen receptor activity. Mol. Endocrinol..

[17] Ciana P (2003). *In vivo* imaging of transcriptionally active estrogen receptors. Nat. Med..

[18] Ciana P (2005). Estrogenic activities in rodent estrogen-free diets. Endocrinology..

[19] Goeman F (2012). Molecular imaging of nuclear Factor-Y transcriptional activity maps proliferation sites in live animals. Mol Biol Cell..

[20] Tkalcević VI, Cuzić S, Parnham MJ, Pasalić I, Brajsa K (2009). Differential evaluation of excisional non-occluded wound healing in db/db mice. Toxicol. Pathol..

