# Supplementary material for: Novel Locally Active Estrogens Accelerate Cutaneous Wound Healing-Part 2
Source: Sci Rep. 2017 May 31;7:2510. doi: 10.1038/s41598-017-02820-y (PMC5451472; doi:10.1038/s41598-017-02820-y)
Supplement: Supplementary file 1 — Supplementary informations [file 41598_2017_2820_MOESM1_ESM.pdf]

Novel Locally Active Estrogens Accelerate Cutaneous Wound Healing-Part 2.

Mario Brufani†+, Nicoletta Rizzi#+, Clara Meda#, Luigi Filocamo†, Francesca Ceccacci*◊, Virginia D'Aiuto§, Gabriele Bartoli§, Angela La Bella§, Luisa M. Migneco§, Rinaldo Marini Bettolo§, Francesca Leonelli◊, Paolo Ciana# and Adriana Maggi*#*

†Dipartimento di Scienze Biochimiche "A. Rossi Fanelli", Universita` degli Studi di Roma "La Sapienza", Via degli Apuli 9, I-00185 Roma, Italy

§Dipartimento di Chimica "S. Cannizzaro" and ◊ Dipartimento di Biologia Ambientale, Universita` degli Studi di Roma "La Sapienza", P.le Aldo Moro 5, I-00185 Roma, Italy

◊ Istituto di Metodologie Chimiche-CNR, Unità Organizzativa di Supporto, Sede di Roma, Università degli Studi di Roma "La Sapienza", P. le Aldo Moro, 5, I-00185 Roma, Italy

#Centre of Excellence on Neurodegenerative Diseases, Universita` degli Studi di Milano, Via Balzaretti, 9, I-20133 Milano, Italy

Table of Contents

^{1}H-NMR, ^{13}C-NMR spectra of new 17β-estradiol derivatives **1**, **6** and **7**

**1a**	pg. S3-S4
**1b**	pg. S5-S6
**1c**	pg. S7-S8
**1d**	pg. S8-S10
**1e**	pg. S11-S12
**1f**	pg. S13-S14
**1g**	pg. S15-S16
**1h**	pg. S17-S18
**6a**	pg. S19-S20
**6b**	pg. S21-S22
**7**	pg. S23-S24

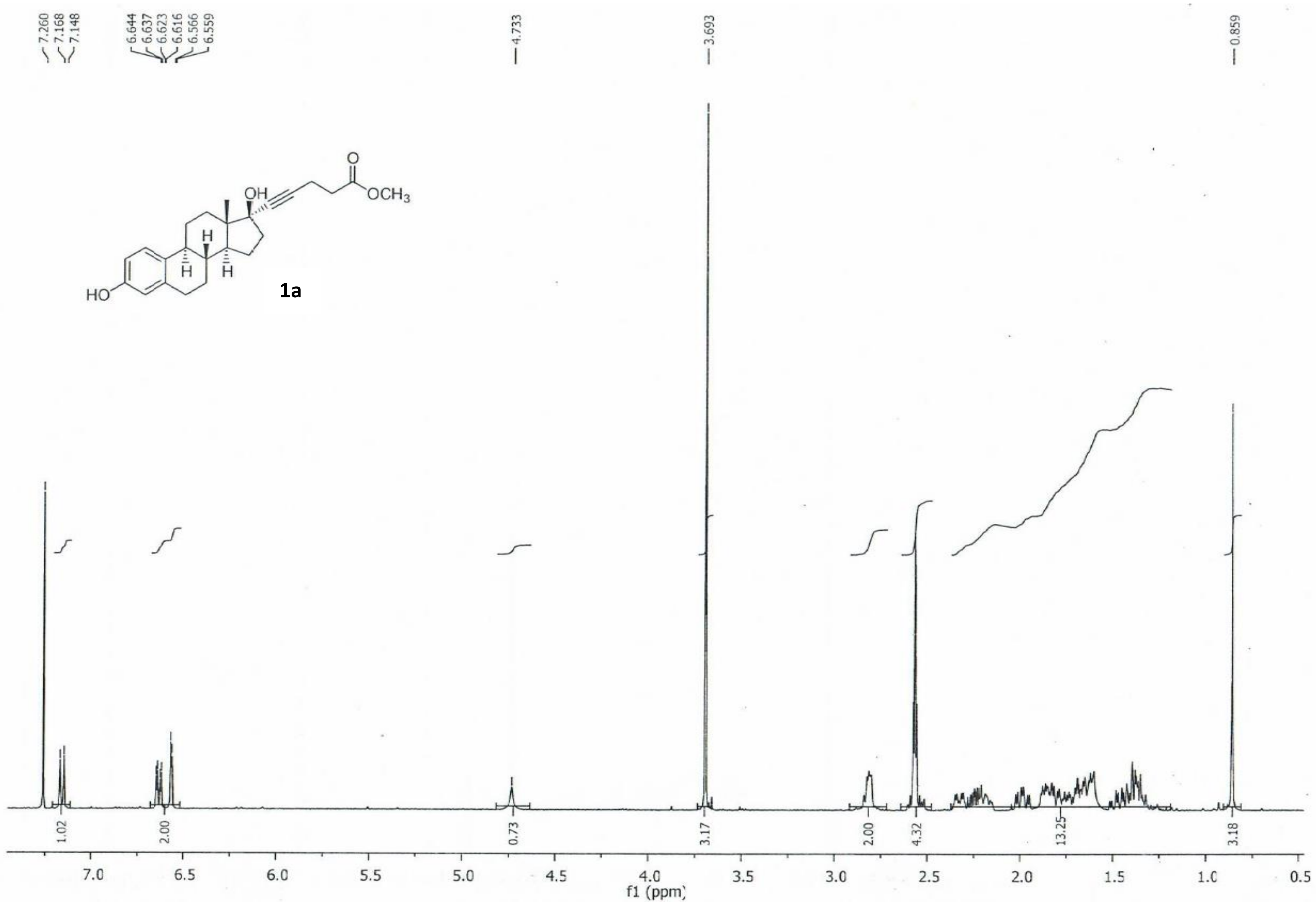
7.260
7.168
7.148
6.644
6.637
6.623
6.616
6.566
6.559
4.733
3.693
0.859
OH
OCH3
HO
H
1a
1.02
2.00
0.73
3.17
2.00
4.32
13.25
3.18
7.0
6.5
6.0
5.5
5.0
4.5
4.0
3.5
3.0
2.5
2.0
1.5
1.0
0.5
f1 (ppm)

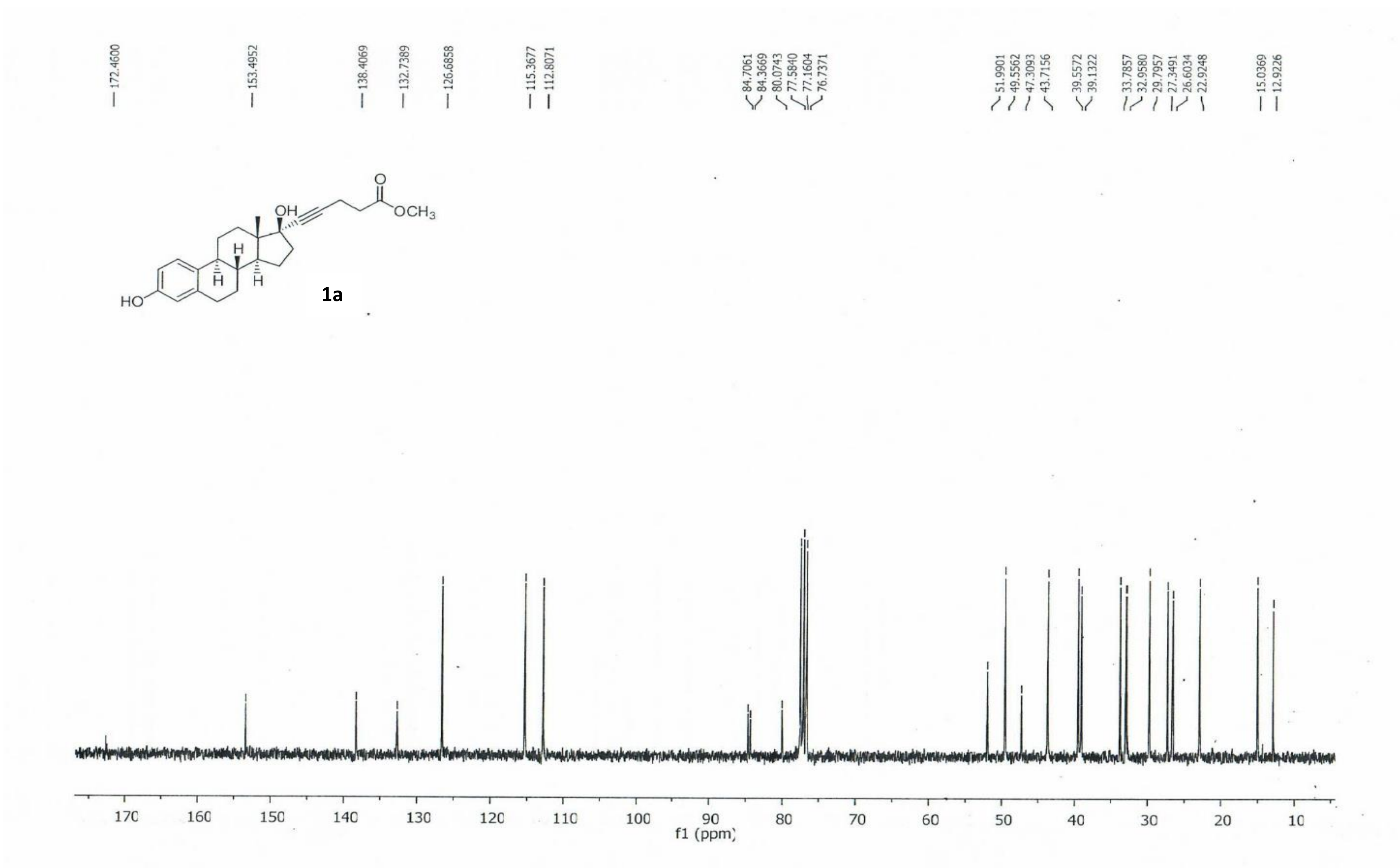
172.4600
153.4952
138.4069
132.7389
126.6858
115.3677
112.8071
84.7061
84.3669
80.0743
77.5840
77.1604
76.7371
51.9901
49.5562
47.3093
43.7156
39.5572
39.1322
33.7857
32.9580
29.7957
27.3491
26.6034
22.9248
15.0369
12.9226
O
OH
OCH3
HO
H
1a
170 160 150 140 130 120 110 100 90 80 70 60 50 40 30 20 10
f1 (ppm)

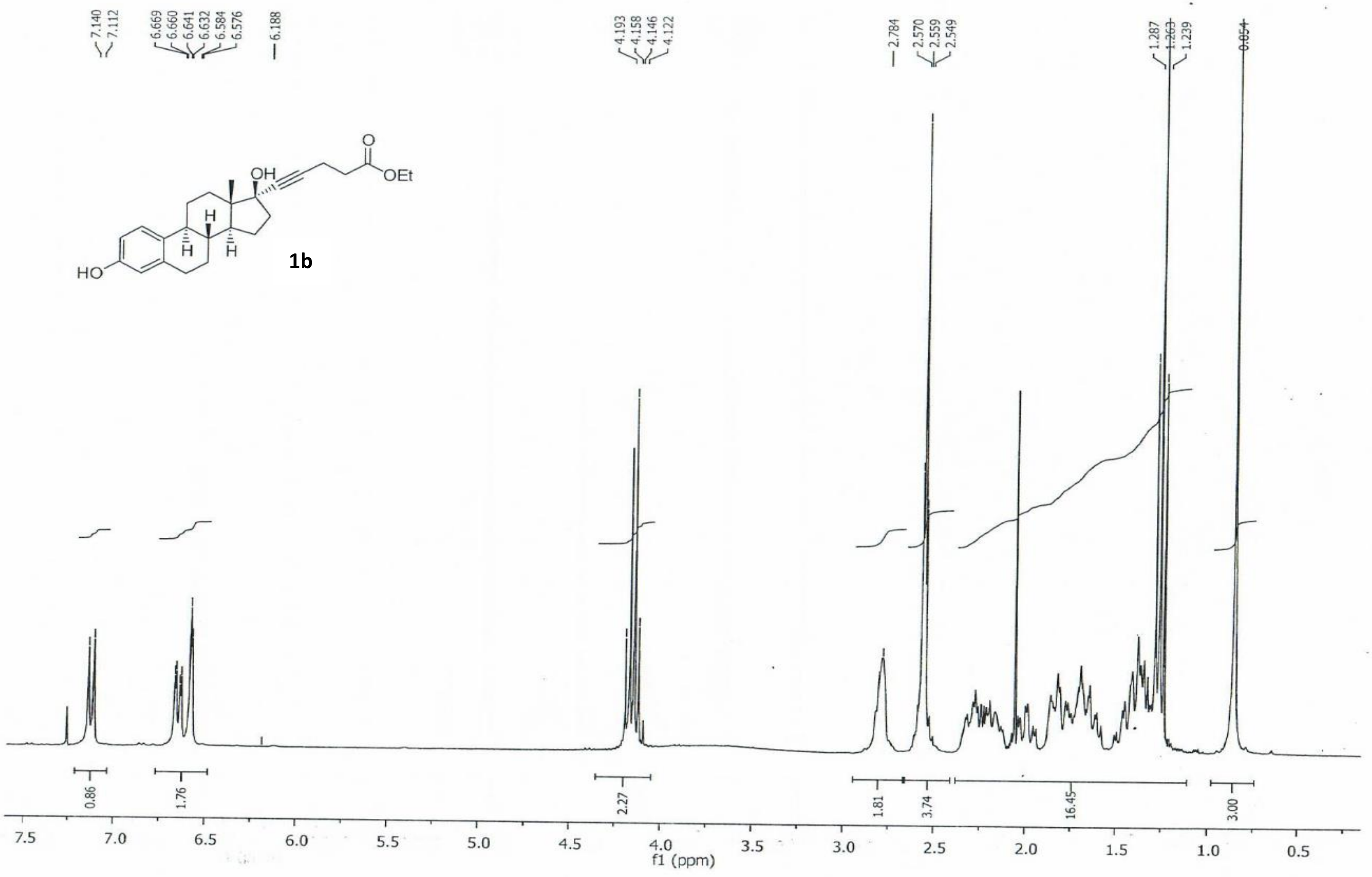

7.140
7.112
6.669
6.660
6.641
6.632
6.584
6.576
6.188
4.193
4.158
4.146
4.122
2.784
2.570
2.559
2.549
1.287
1.263
1.239
0.854
OH
O
OEt
H
HO
0.86
1.76
2.27
1.81
3.74
16.45
3.00
f1 (ppm)

1b

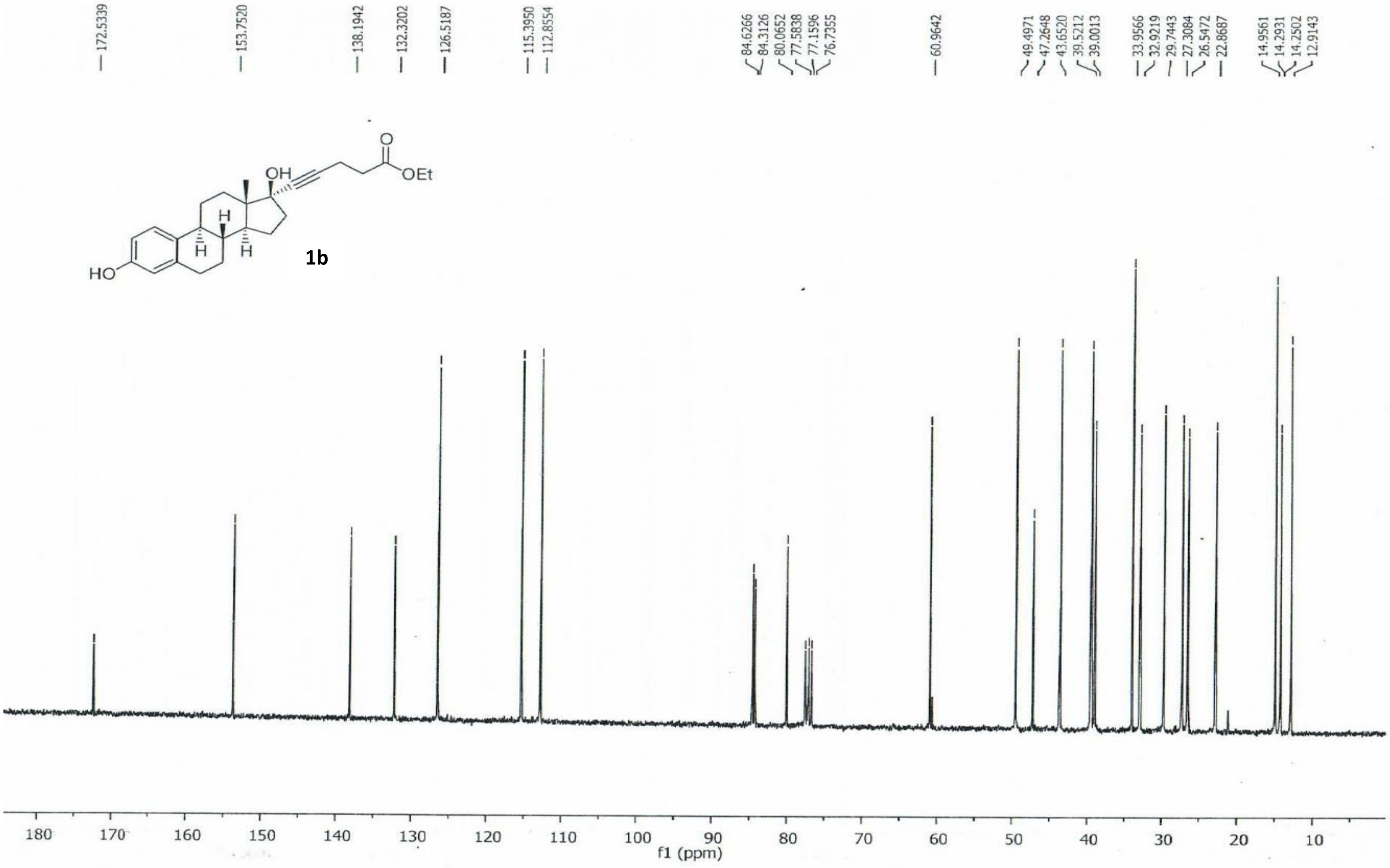
172.5339
153.7520
138.1942
132.3202
126.5187
115.3950
112.8554
84.6266
84.3126
80.0652
77.5838
77.1596
76.7355
60.9642
49.4971
47.2648
43.6520
39.5212
39.0013
33.9566
32.9219
29.7443
27.3084
26.5472
22.8687
14.9561
14.2931
14.2502
12.9143
OH
O
OEt
H
HO
1b
180 170 160 150 140 130 120 110 100 90 80 70 60 50 40 30 20 10
f1 (ppm)

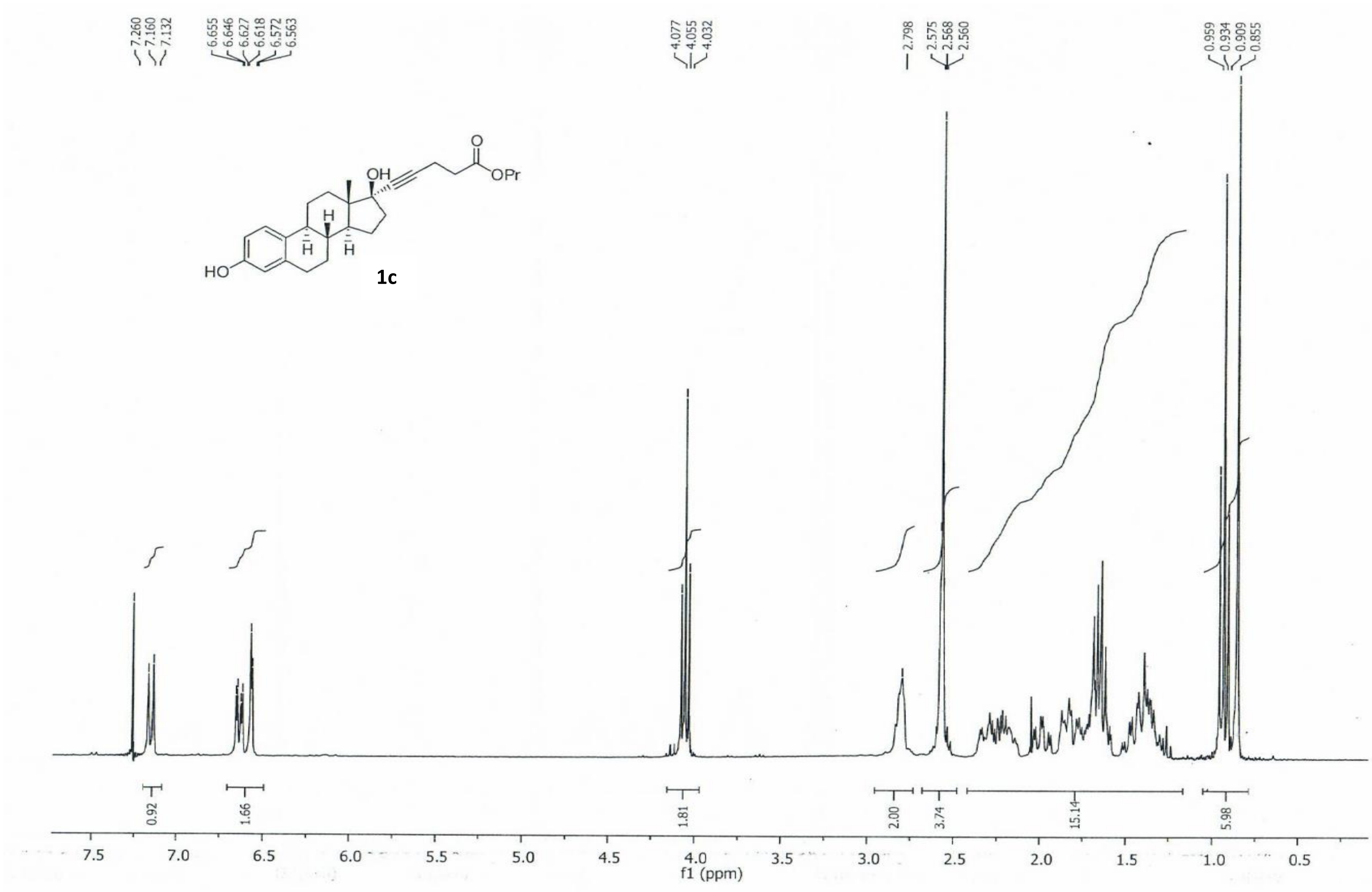
7.260
7.160
7.132
6.655
6.646
6.627
6.618
6.572
6.563
4.077
4.055
4.032
2.798
2.575
2.568
2.560
0.959
0.934
0.909
0.855
OH
OPr
O
H
H
H
HO
1c
0.92
1.66
1.81
2.00
3.74
15.14
5.98
7.5
7.0
6.5
6.0
5.5
5.0
4.5
4.0
3.5
3.0
2.5
2.0
1.5
1.0
0.5
f1 (ppm)

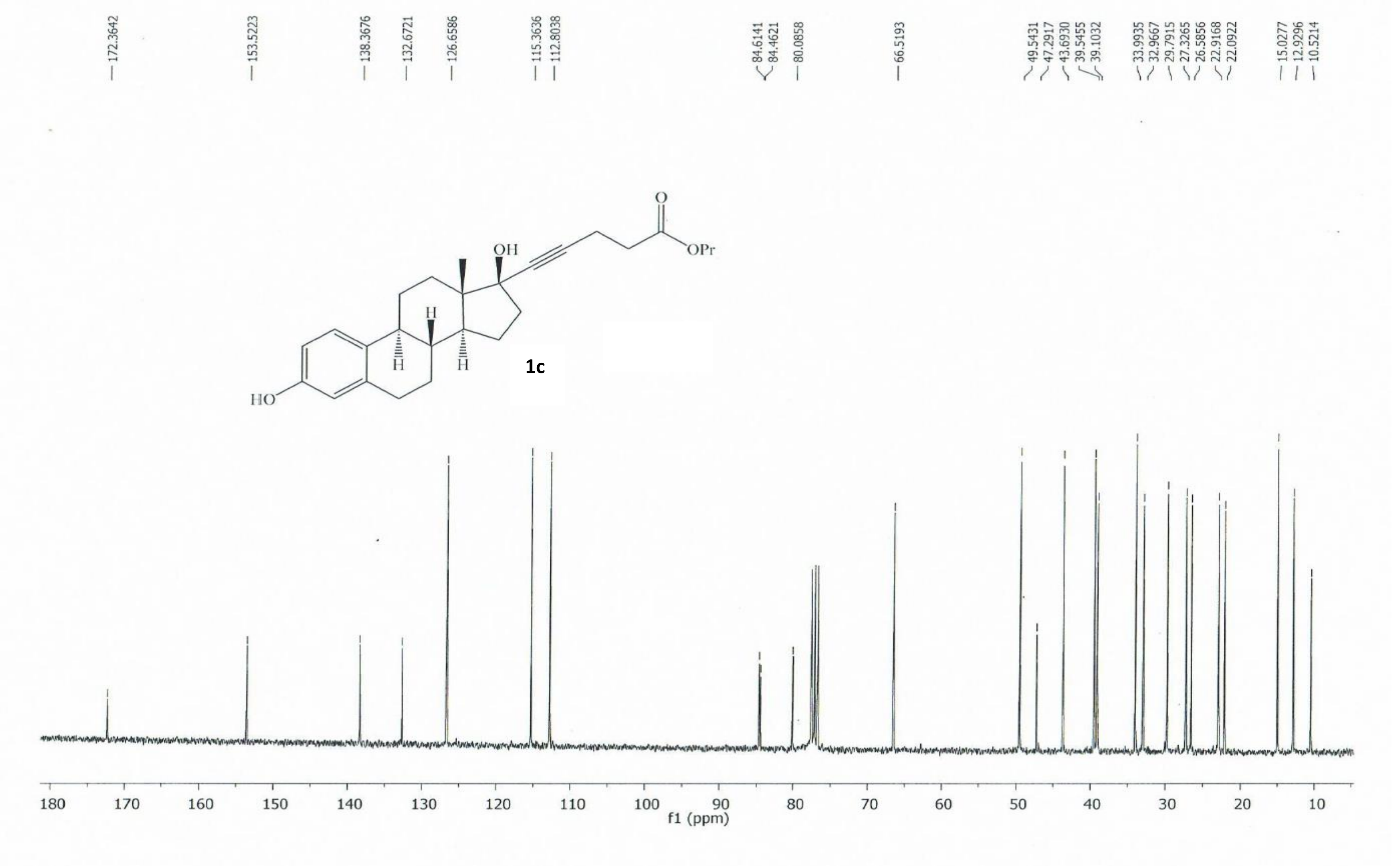
172.3642
153.5223
138.3676
132.6721
126.6586
115.3636
112.8038
84.6141
84.4621
80.0858
66.5193
49.5431
47.2917
43.6930
39.5455
39.1032
33.9935
32.9667
29.7915
27.3265
26.5856
22.9168
22.0922
15.0277
12.9296
10.5214
O
OH
OPr
H
H
H
HO
1c
180
170
160
150
140
130
120
110
100
90
80
70
60
50
40
30
20
10
f1 (ppm)

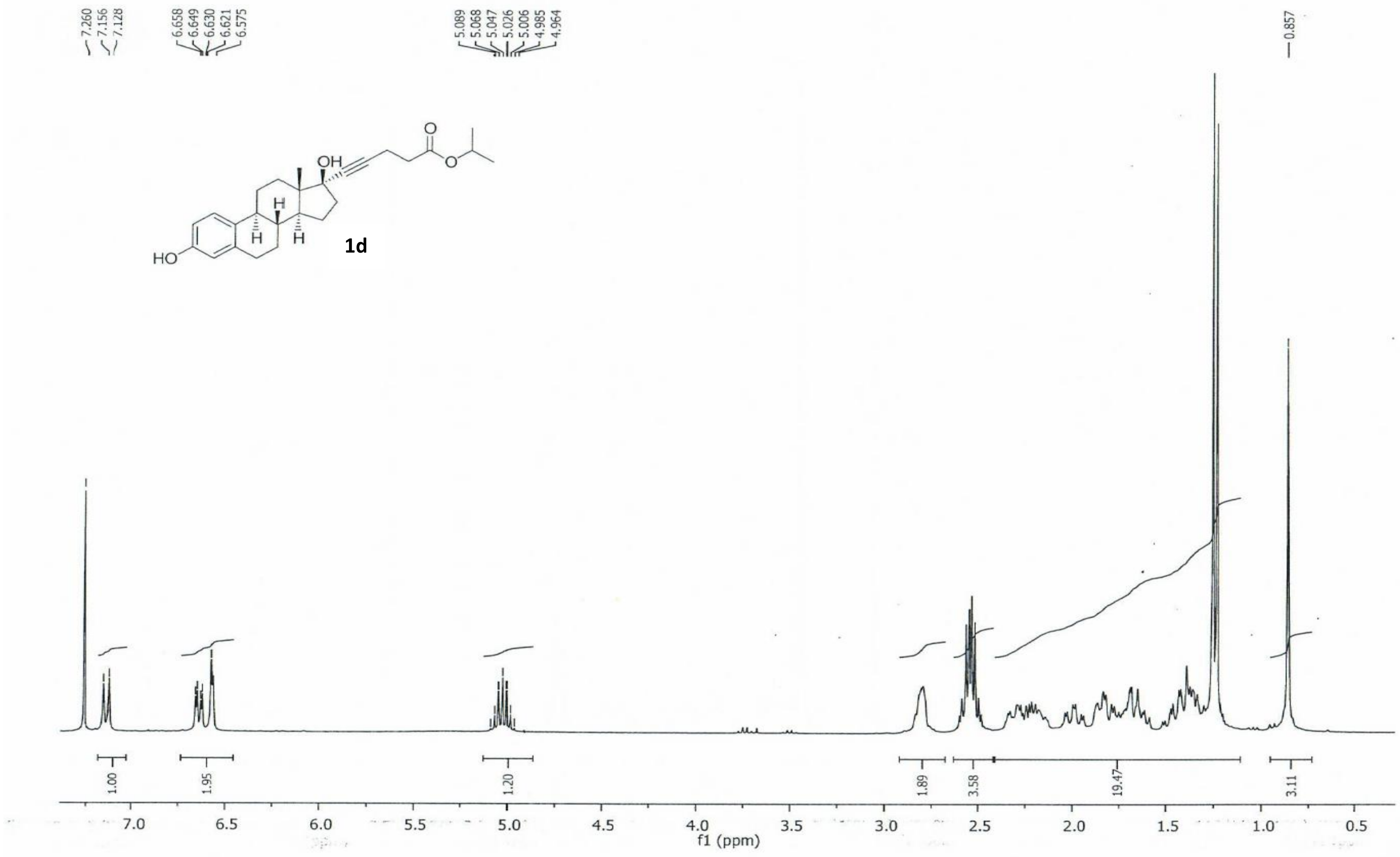
7.260
7.156
7.128
6.658
6.649
6.630
6.621
6.575
5.089
5.068
5.047
5.026
5.006
4.985
4.964
0.857
OH
HO
H
H
H
O
O
1d
1.00
1.95
1.20
1.89
3.58
19.47
3.11
7.0
6.5
6.0
5.5
5.0
4.5
4.0
3.5
3.0
2.5
2.0
1.5
1.0
0.5
f1 (ppm)

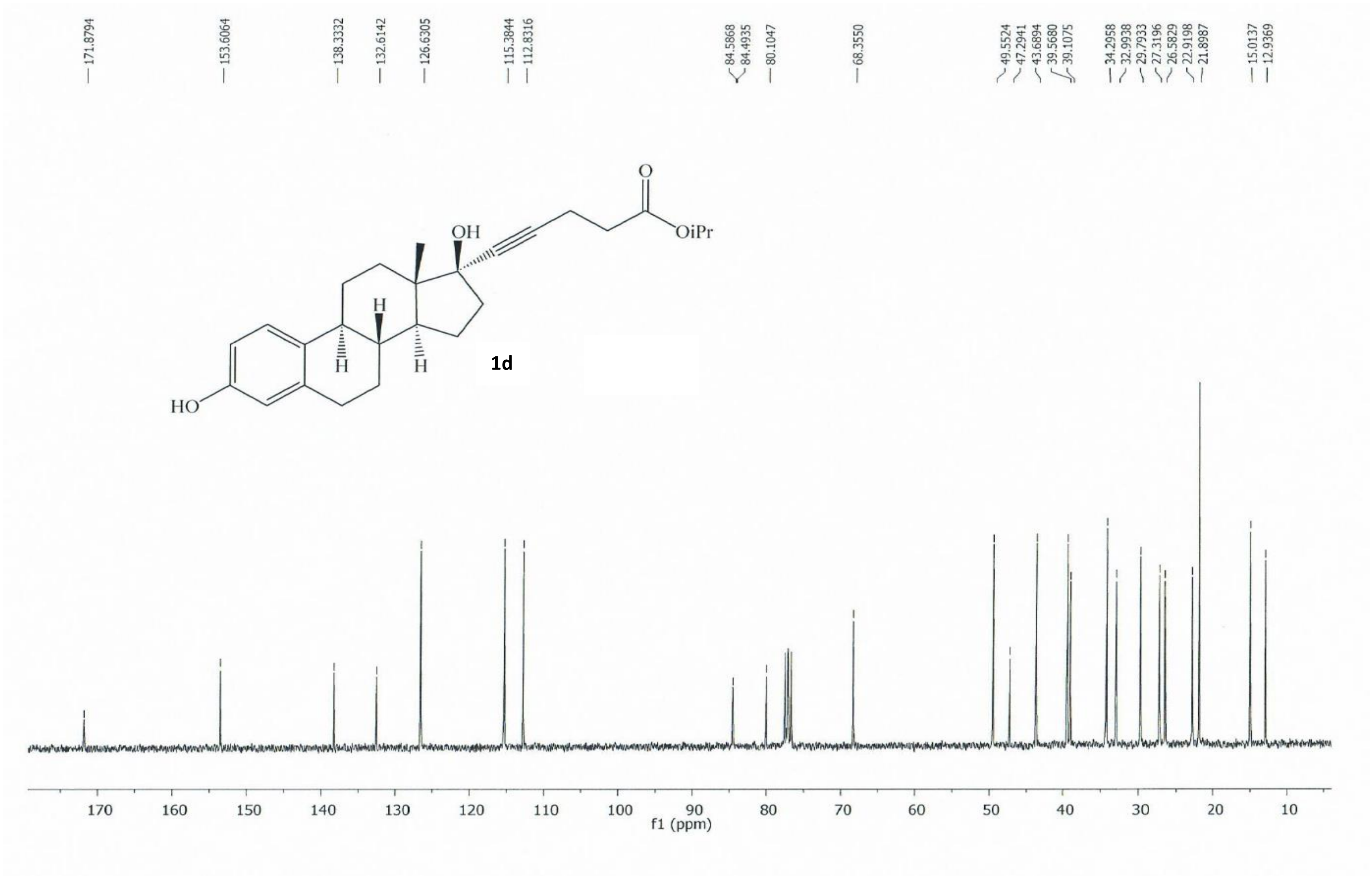
171.8794
153.6064
138.3332
132.6142
126.6305
115.3844
112.8316
84.5868
84.4935
80.1047
68.3550
49.5524
47.2941
43.6894
39.5680
39.1075
34.2958
32.9938
29.7933
27.3196
26.5829
22.9198
21.8987
15.0137
12.9369
OH
O
OiPr
H
H
H
HO
f1 (ppm)

1d

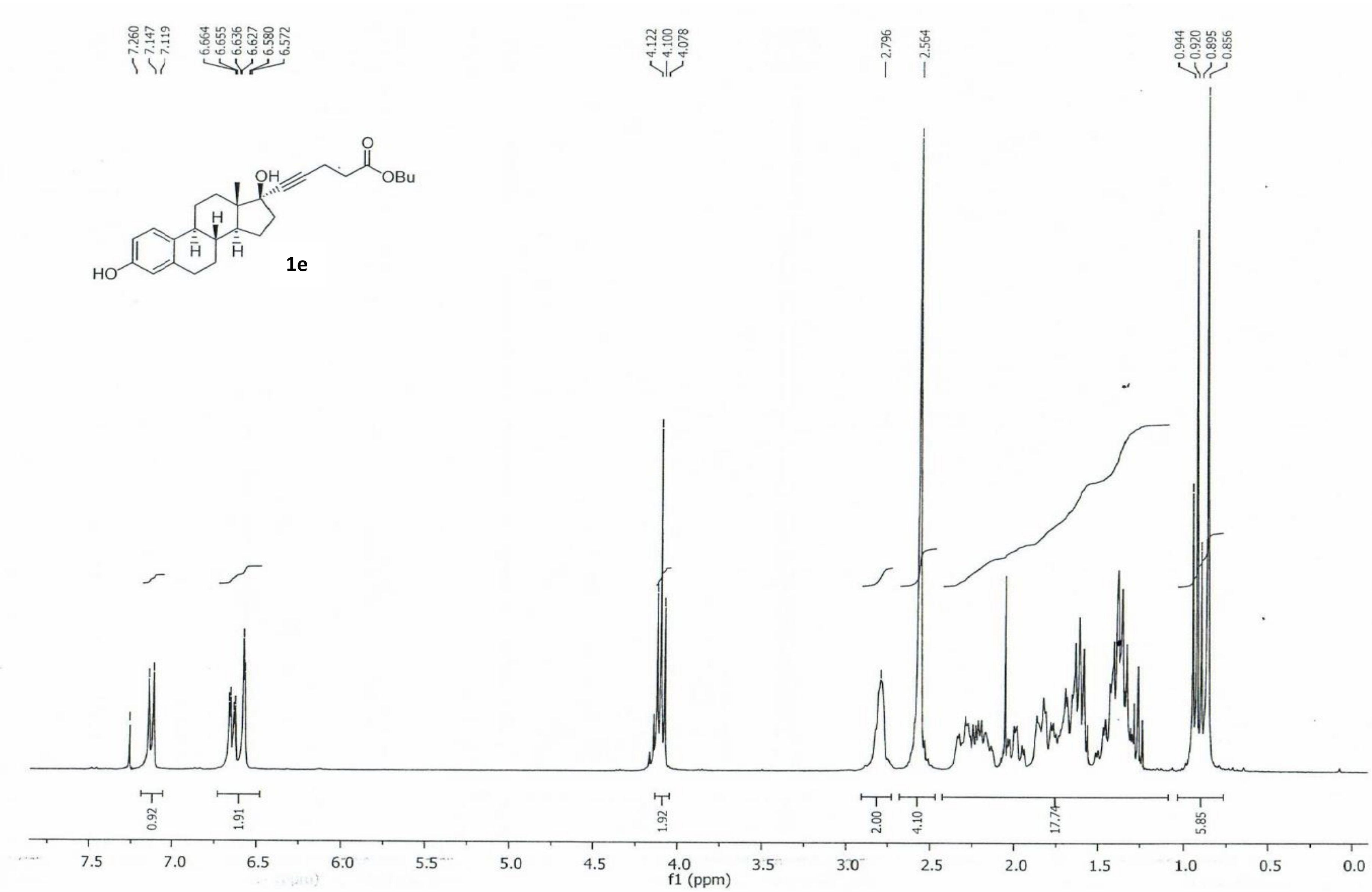

7.260
7.147
7.119
6.664
6.655
6.636
6.627
6.580
6.572
4.122
4.100
4.078
2.796
2.564
0.944
0.920
0.895
0.856
OH
O
OBu
H
H
H
HO
1e
0.92
1.91
1.92
2.00
4.10
17.74
5.85
7.5
7.0
6.5
6.0
5.5
5.0
4.5
4.0
3.5
3.0
2.5
2.0
1.5
1.0
0.5
0.0
f1 (ppm)

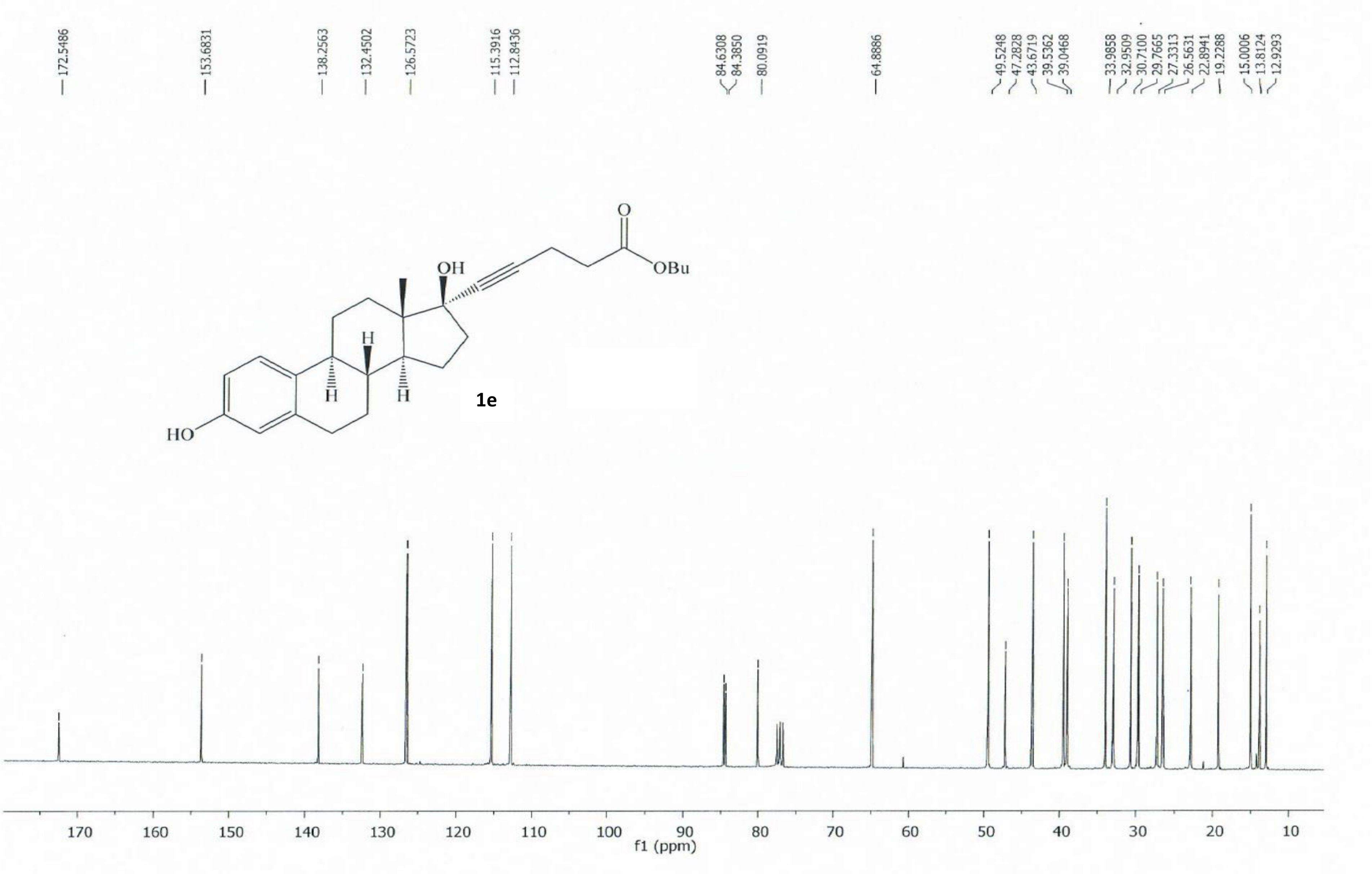
172.5486
153.6831
138.2563
132.4502
126.5723
115.3916
112.8436
84.6308
84.3850
80.0919
64.8886
49.5248
47.2828
43.6719
39.5362
39.0468
33.9858
32.9509
30.7100
29.7665
27.3313
26.5631
22.8941
19.2288
15.0006
13.8124
12.9293
OH
O
OBu
H
H
H
HO
1e
170 160 150 140 130 120 110 100 90 80 70 60 50 40 30 20 10
f1 (ppm)

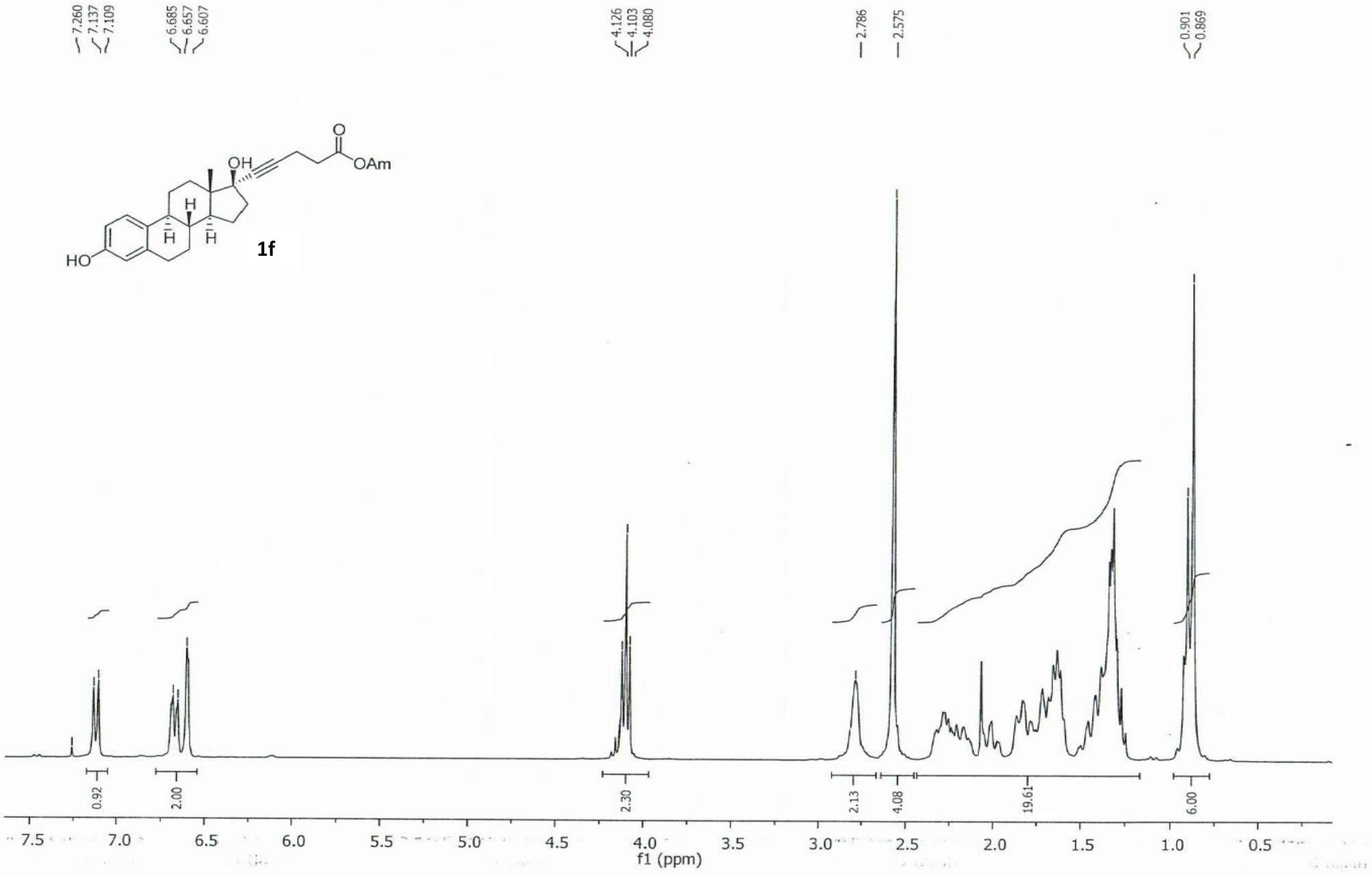
7.260
7.137
7.109
6.685
6.657
6.607
4.126
4.103
4.080
2.786
2.575
0.901
0.869
OH
OAm
HO
H
1f
0.92
2.00
2.30
2.13
4.08
19.61
6.00
7.5
7.0
6.5
6.0
5.5
5.0
4.5
4.0
3.5
3.0
2.5
2.0
1.5
1.0
0.5
f1 (ppm)

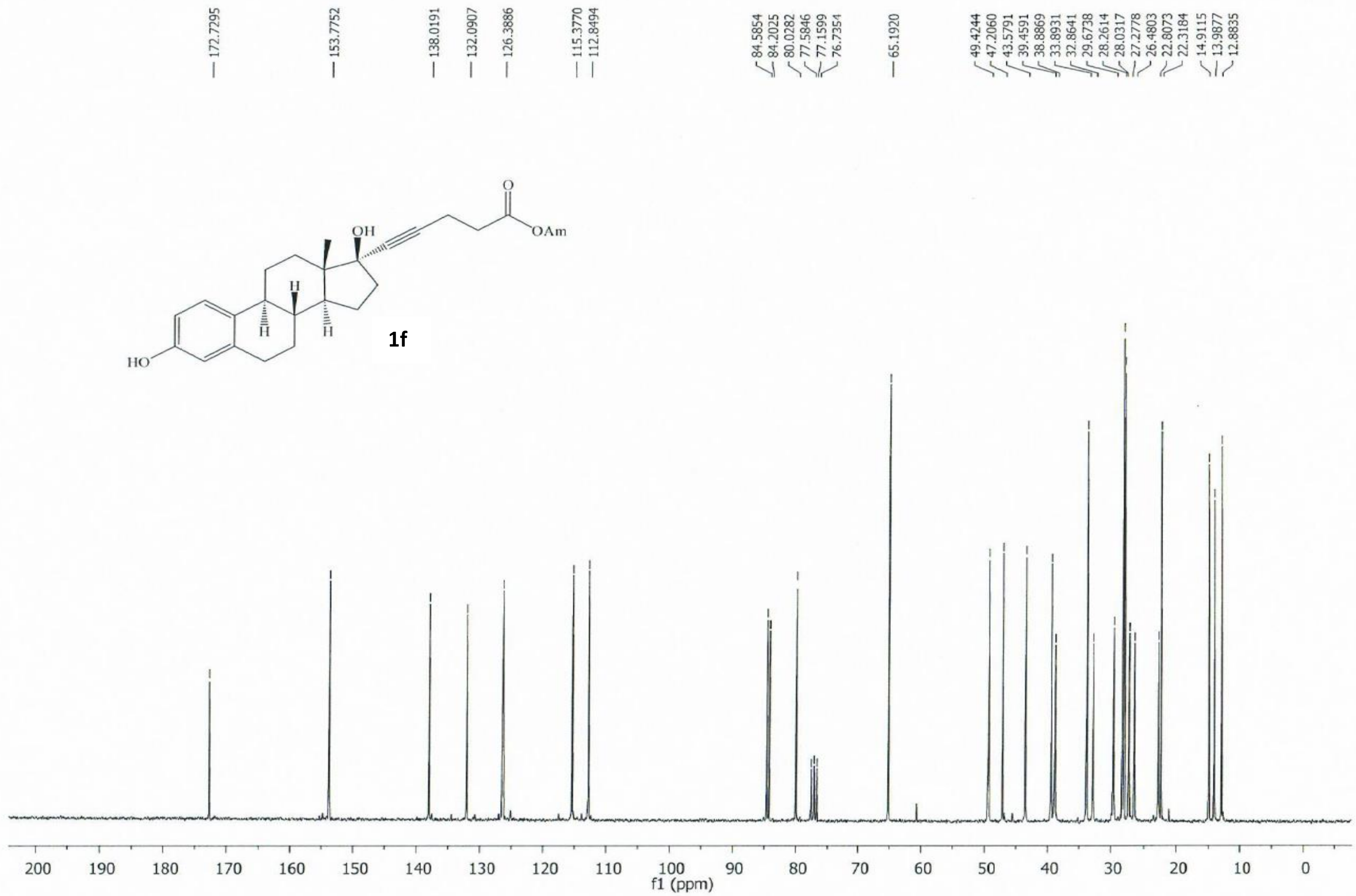
172.7295
153.7752
138.0191
132.0907
126.3886
115.3770
112.8494
84.5854
84.2025
80.0282
77.5846
77.1599
76.7354
65.1920
49.4244
47.2060
43.5791
39.4591
38.8869
33.8931
32.8641
29.6738
28.2614
28.0317
27.2778
26.4803
22.8073
22.3184
14.9115
13.9877
12.8835
O
OH
OAm
H
H
H
HO
1f
200 190 180 170 160 150 140 130 120 110 100 90 80 70 60 50 40 30 20 10 0
f1 (ppm)

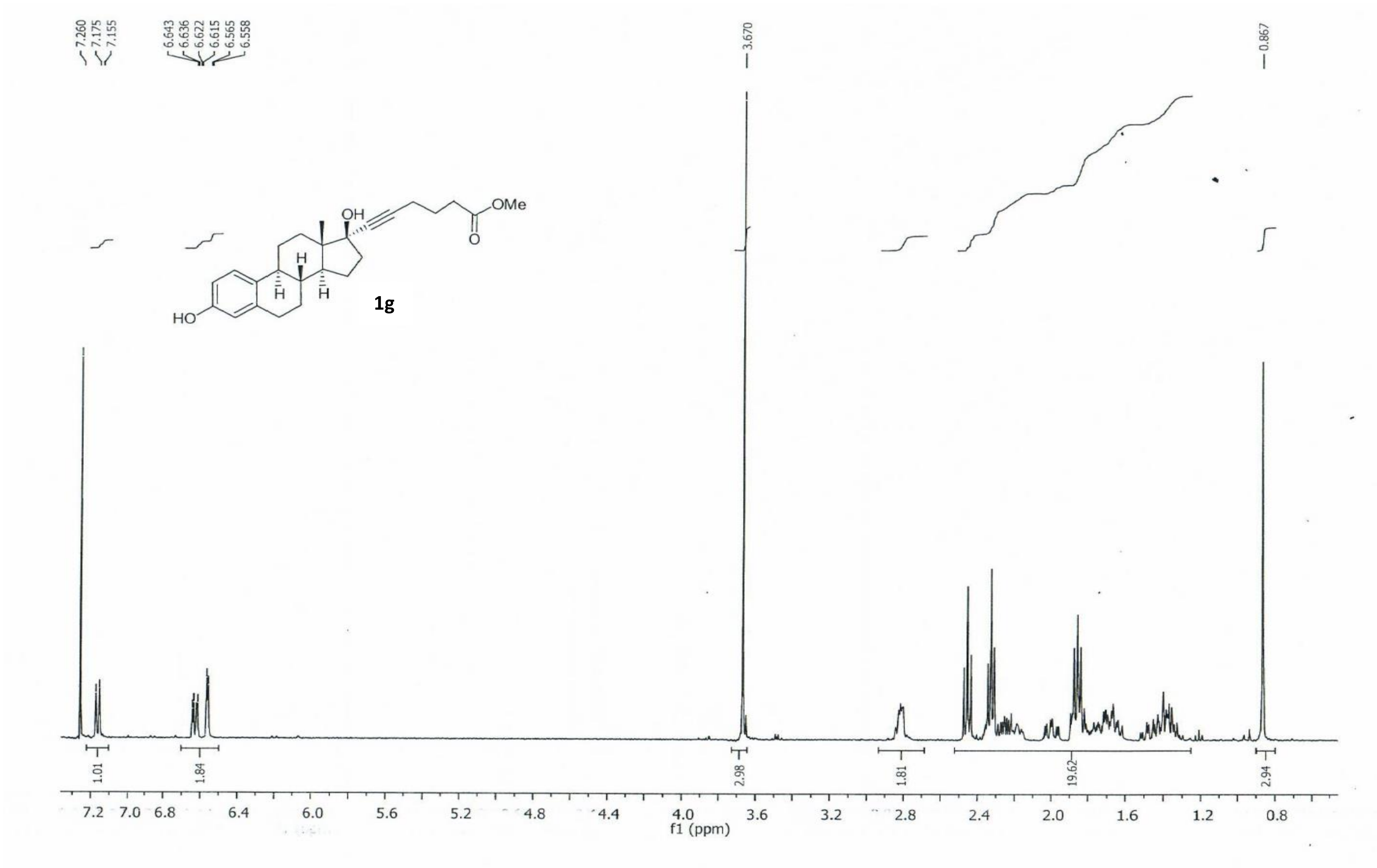
7.260
7.175
7.155
6.643
6.636
6.622
6.615
6.565
6.558
3.670
0.867
OH
OMe
O
H
H
H
HO
1g
1.01
1.84
2.98
1.81
19.62
2.94
7.2
7.0
6.8
6.4
6.0
5.6
5.2
4.8
4.4
4.0
3.6
3.2
2.8
2.4
2.0
1.6
1.2
0.8
f1 (ppm)

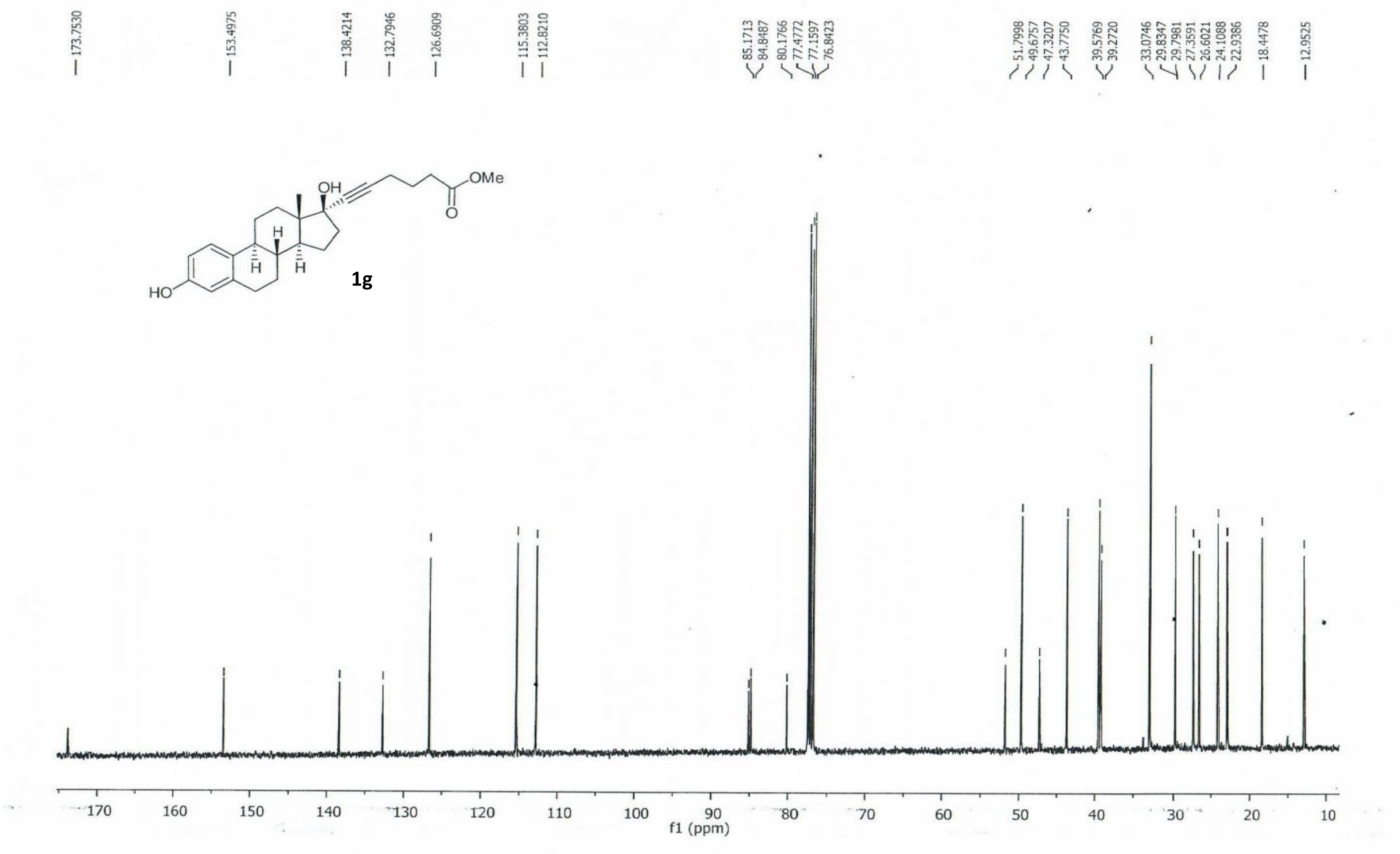
173.7530
153.4975
138.4214
132.7946
126.6909
115.3803
112.8210
85.1713
84.8487
80.1766
77.4772
77.1597
76.8423
51.7998
49.6757
47.3207
43.7750
39.5769
39.2720
33.0746
29.8347
29.7981
27.3591
26.6021
24.1088
22.9386
18.4478
12.9525
OH
OMe
O
H
HO
1g
170
160
150
140
130
120
110
100
90
80
70
60
50
40
30
20
10
f1 (ppm)

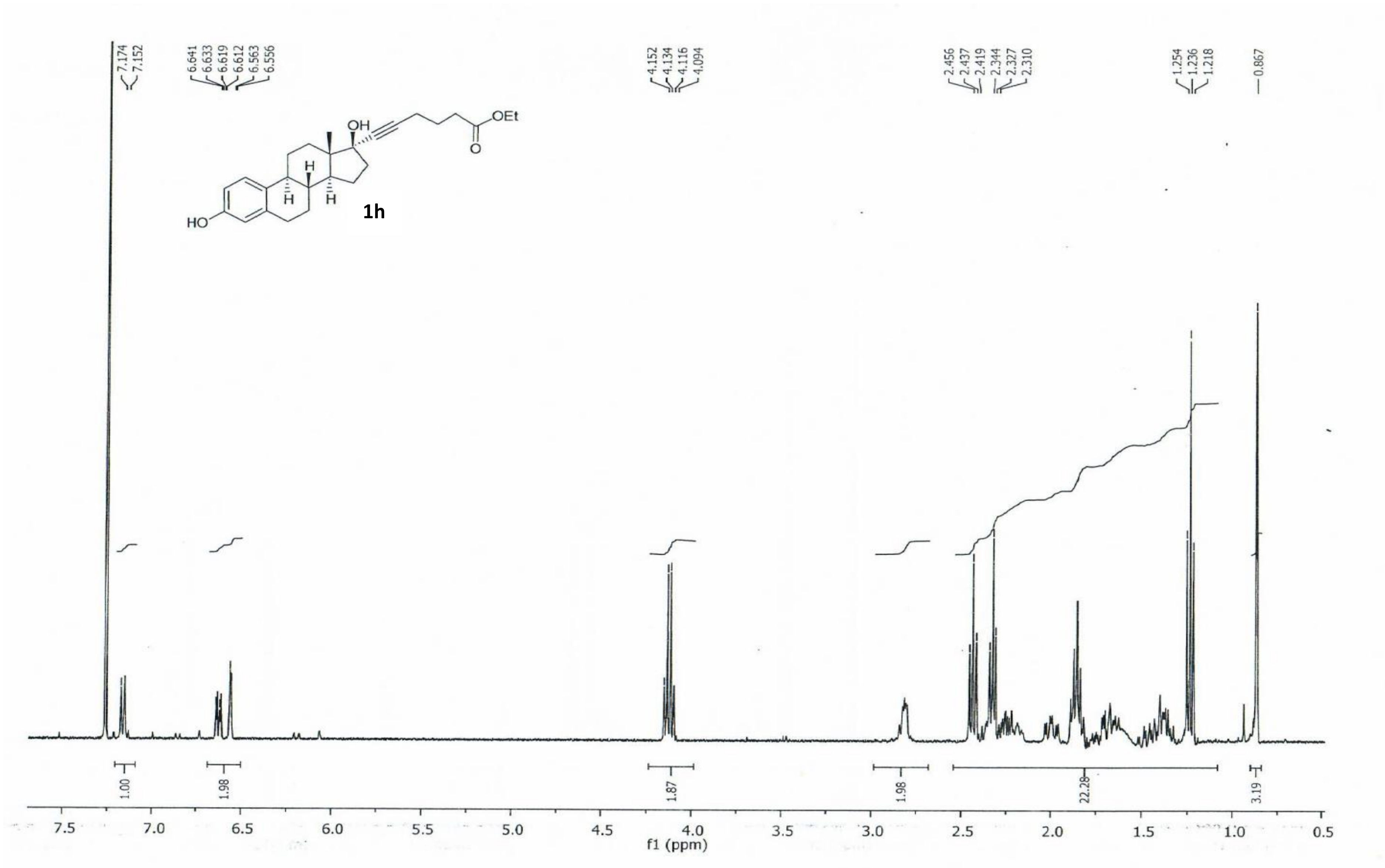
7.174
7.152
6.641
6.633
6.619
6.612
6.563
6.556
4.152
4.134
4.116
4.094
2.456
2.437
2.419
2.344
2.327
2.310
1.254
1.236
1.218
0.867
OH
OEt
O
H
HO
1h
1.00
1.98
1.87
1.98
22.28
3.19
7.5
7.0
6.5
6.0
5.5
5.0
4.5
4.0
3.5
3.0
2.5
2.0
1.5
1.0
0.5
f1 (ppm)

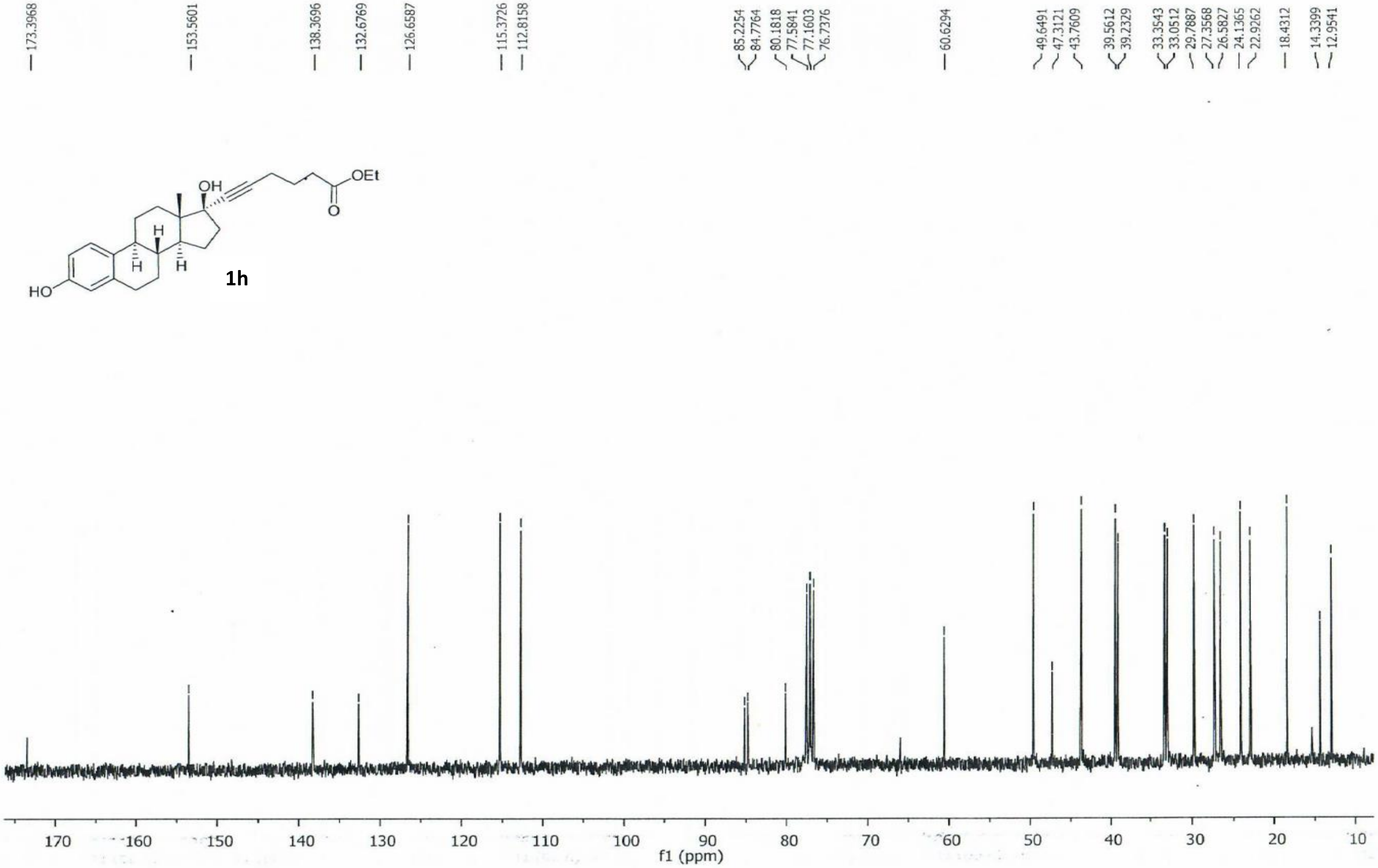
173.3968
153.5601
138.3696
132.6769
126.6587
115.3726
112.8158
85.2254
84.7764
80.1818
77.5841
77.1603
76.7376
60.6294
49.6491
47.3121
43.7609
39.5612
39.2329
33.3543
33.0512
29.7887
27.3568
26.5827
24.1365
22.9262
18.4312
14.3399
12.9541
OH
OEt
O
H
HO
1h
f1 (ppm)

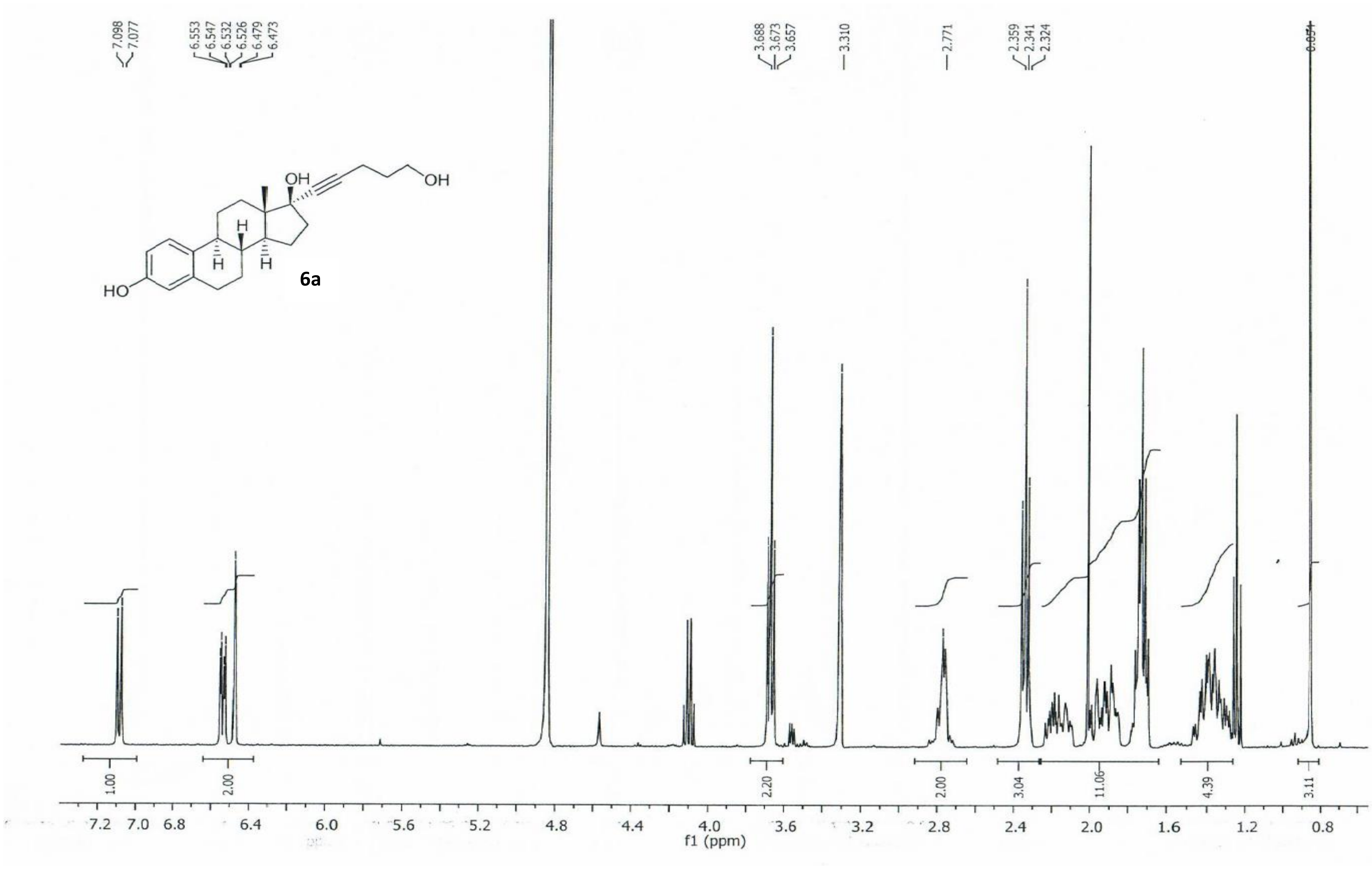
7.098
7.077
6.553
6.547
6.532
6.526
6.479
6.473
3.688
3.673
3.657
3.310
2.771
2.359
2.341
2.324
0.874
OH
OH
H
H
H
HO
6a
1.00
2.00
2.20
2.00
3.04
11.06
4.39
3.11
7.2 7.0 6.8 6.4 6.0 5.6 5.2 4.8 4.4 4.0 3.6 3.2 2.8 2.4 2.0 1.6 1.2 0.8
f1 (ppm)

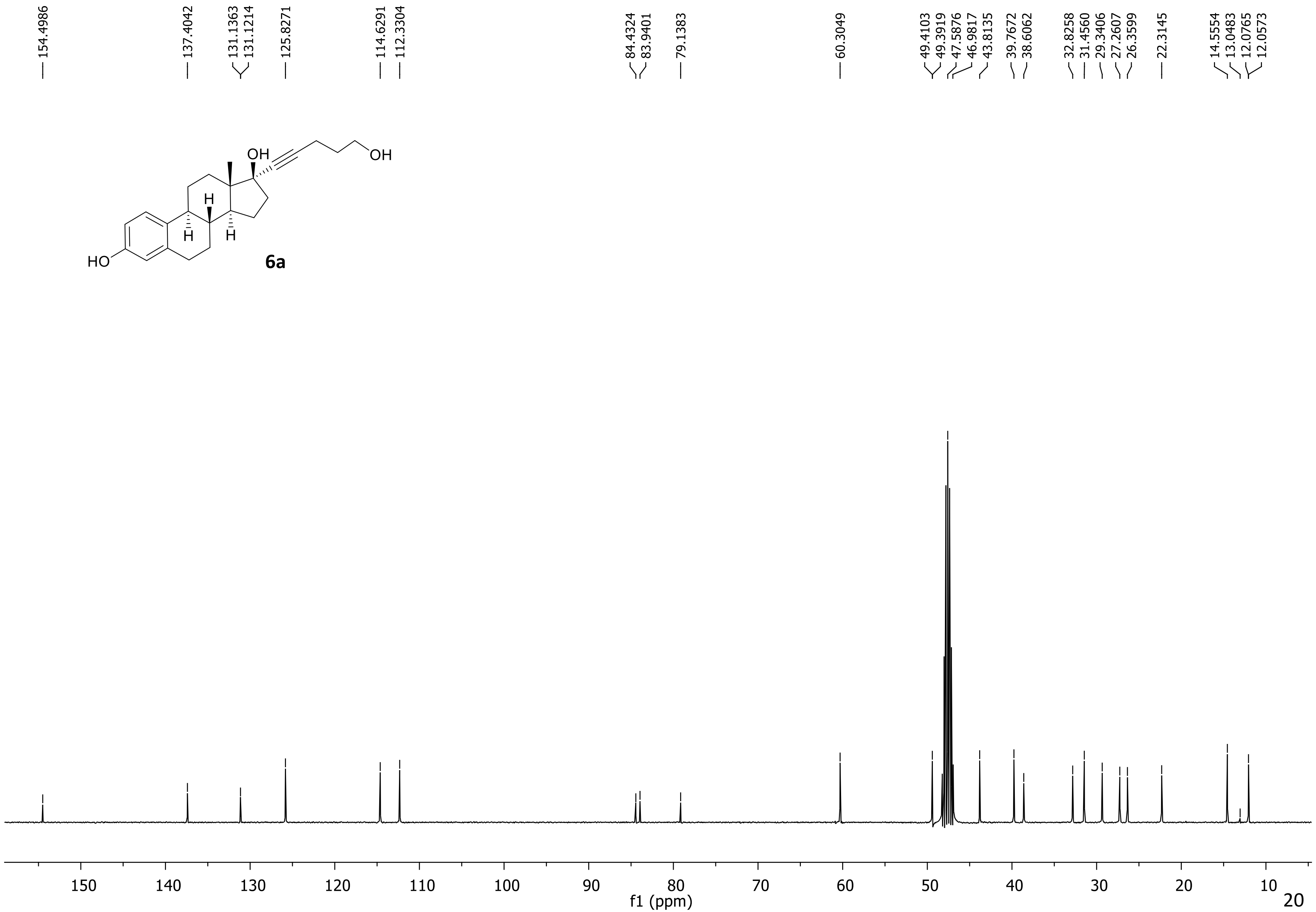
154.4986
137.4042
131.1363
131.1214
125.8271
114.6291
112.3304
84.4324
83.9401
79.1383
60.3049
49.4103
49.3919
47.5876
46.9817
43.8135
39.7672
38.6062
32.8258
31.4560
29.3406
27.2607
26.3599
22.3145
14.5554
13.0483
12.0765
12.0573
OH
OH
H
H
H
HO
6a
150
140
130
120
110
100
90
80
70
60
50
40
30
20
10
f1 (ppm)

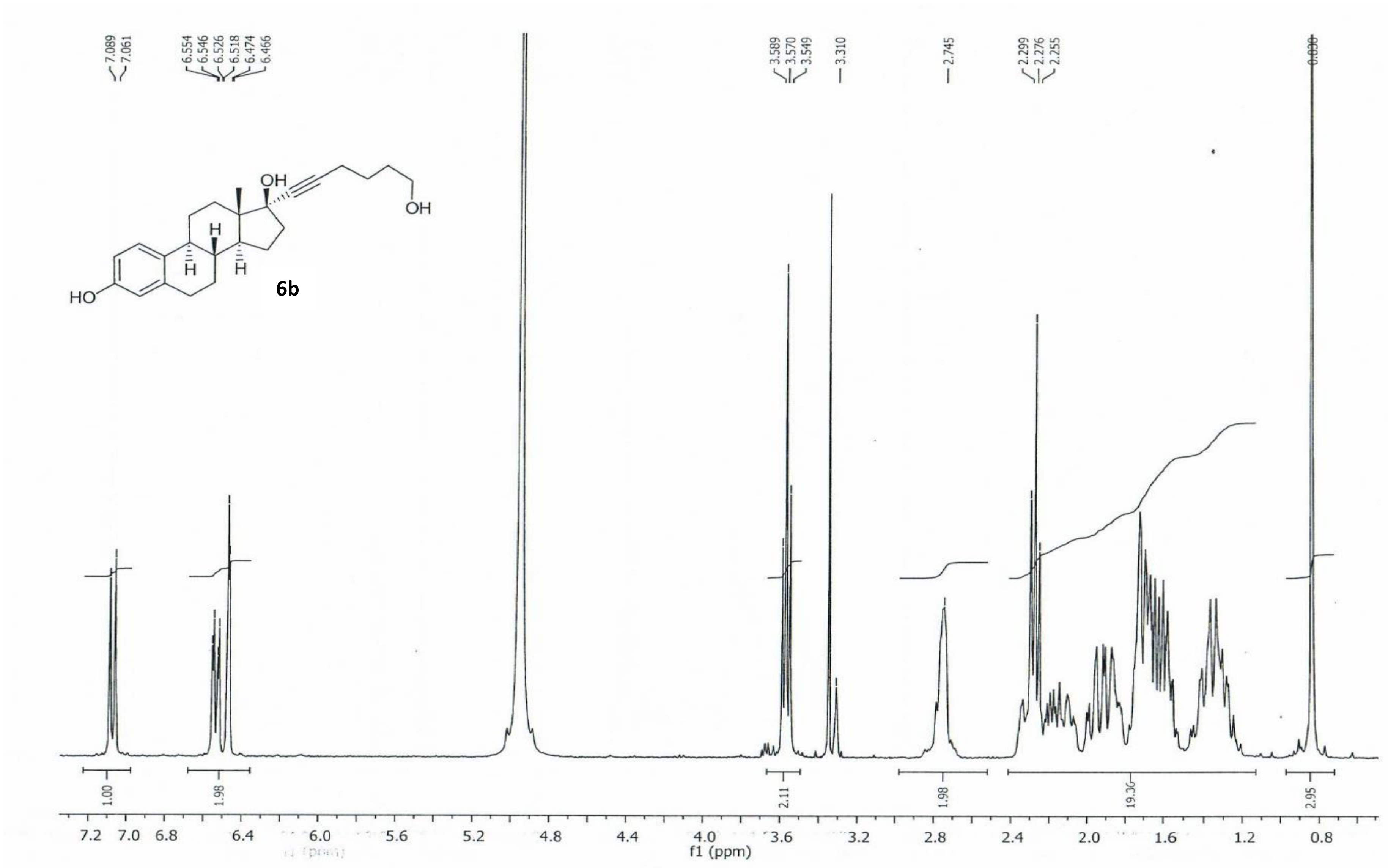
7.089
7.061
6.554
6.546
6.526
6.518
6.474
6.466
3.589
3.570
3.549
3.310
2.745
2.299
2.276
2.255
0.830
OH
OH
H
H
H
HO
6b
1.00
1.98
2.11
1.98
19.30
2.95
7.2
7.0
6.8
6.4
6.0
5.6
5.2
4.8
4.4
4.0
3.6
3.2
2.8
2.4
2.0
1.6
1.2
0.8
f1 (ppm)

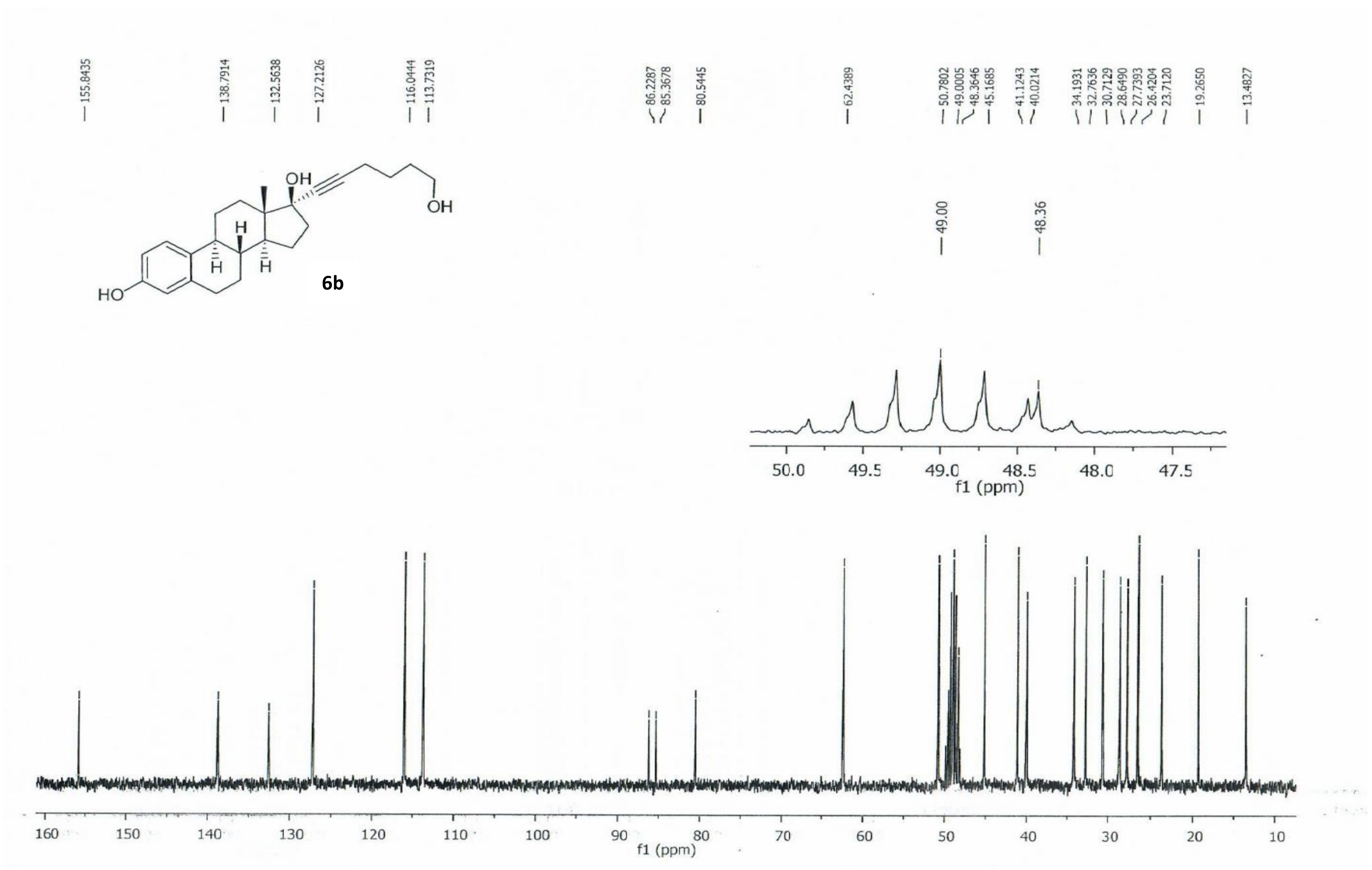
155.8435
138.7914
132.5638
127.2126
116.0444
113.7319
86.2287
85.3678
80.5445
62.4389
50.7802
49.0005
48.3646
45.1685
41.1243
40.0214
34.1931
32.7636
30.7129
28.6490
27.7393
26.4204
23.7120
19.2650
13.4827
OH
OH
HO
H
H
H
6b
49.00
48.36
50.0 49.5 49.0 48.5 48.0 47.5
f1 (ppm)
160 150 140 130 120 110 100 90 80 70 60 50 40 30 20 10
f1 (ppm)

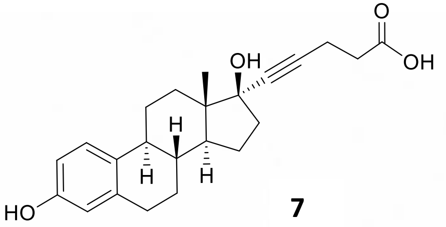

7

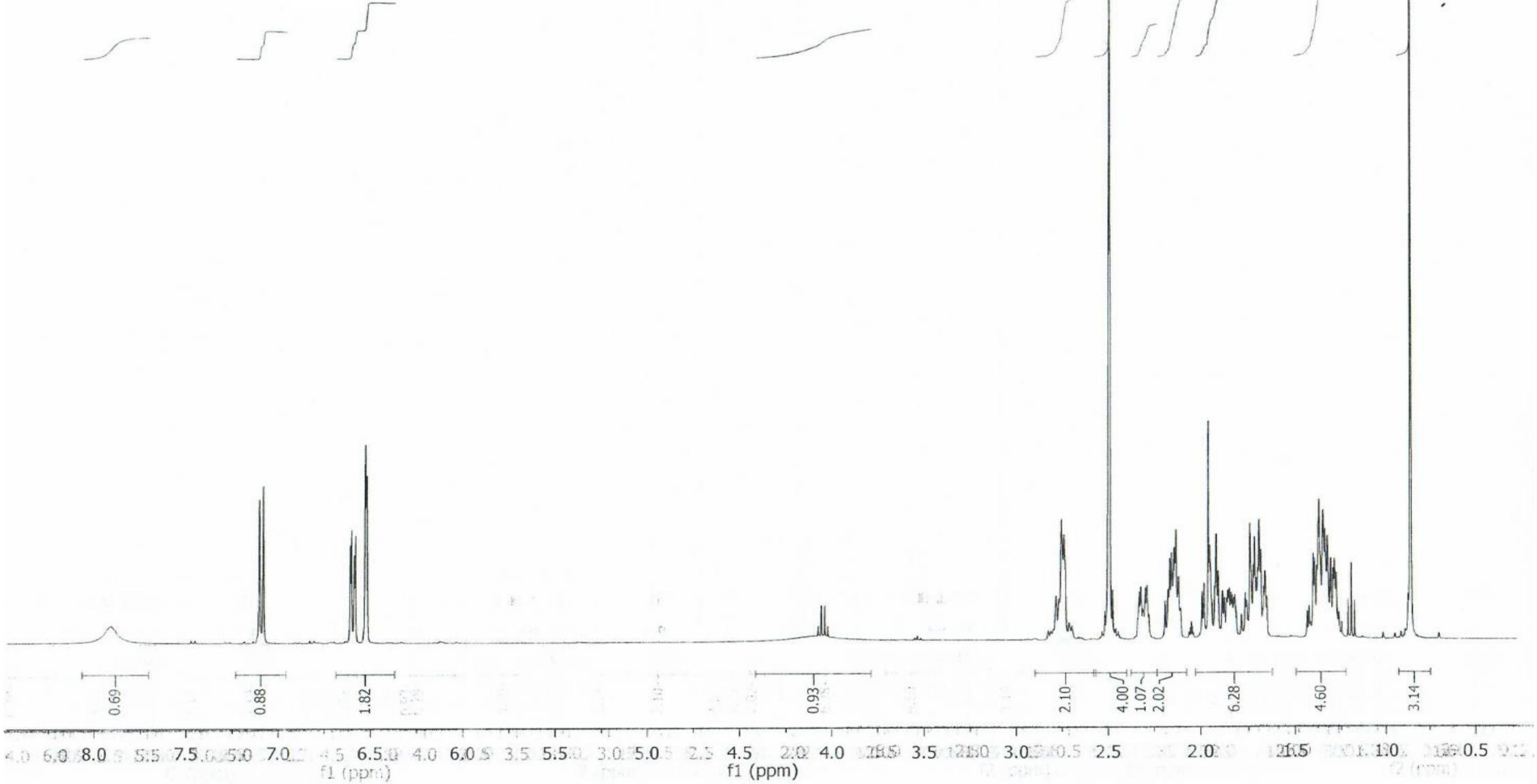

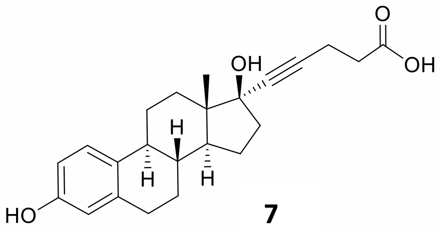
OH
O
OH
H
H
H
HO

7

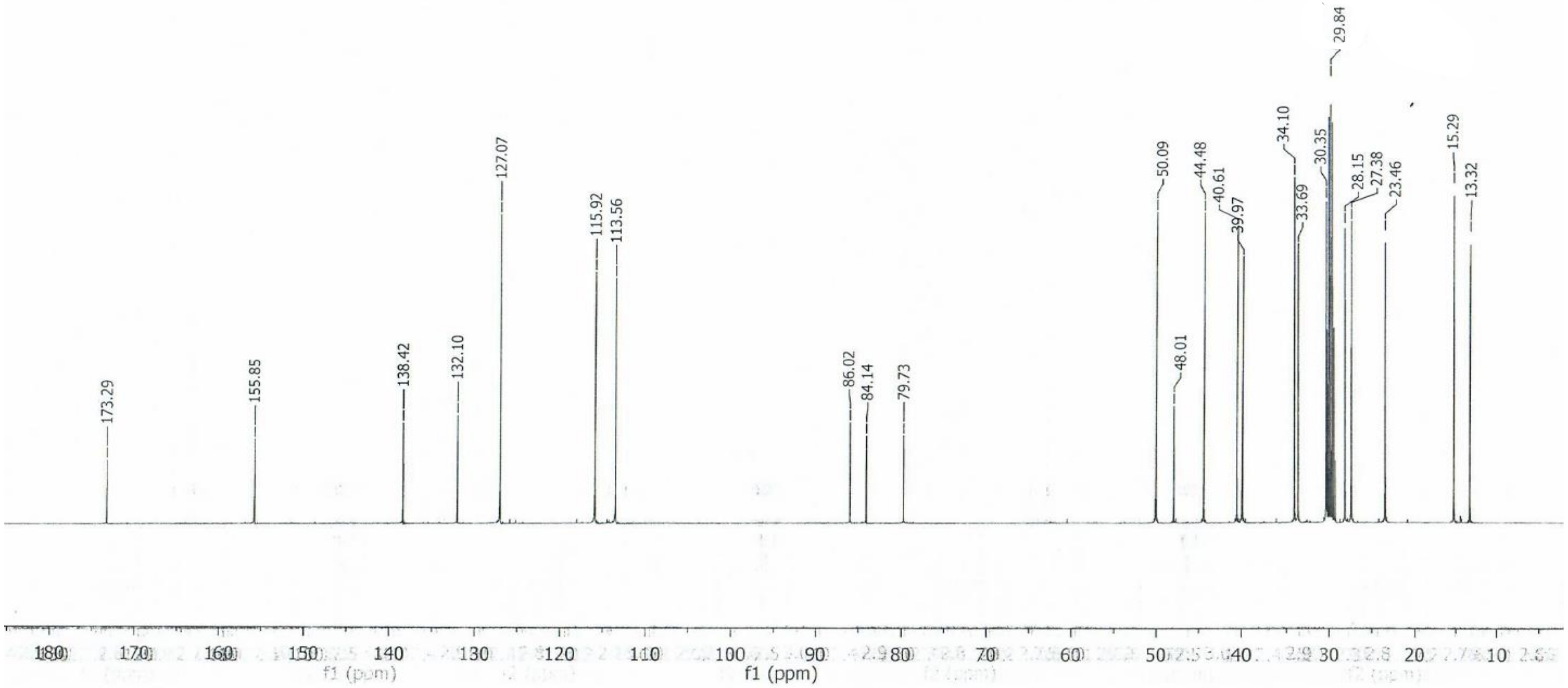
173.29
155.85
138.42
132.10
127.07
115.92
113.56
86.02
84.14
79.73
50.09
48.01
44.48
40.61
39.97
34.10
33.69
30.35
29.84
28.15
27.38
23.46
15.29
13.32
180
170
160
150
140
130
120
110
100
90
80
70
60
50
40
30
20
10
f1 (ppm)
